# Molecular interactions between monoclonal oligomer-specific antibody 5E3 and its amyloid beta cognates

**DOI:** 10.1371/journal.pone.0232266

**Published:** 2020-05-29

**Authors:** Massih Khorvash, Nick Blinov, Carol Ladner-Keay, Jie Lu, Judith M. Silverman, Ebrima Gibbs, Yu Tian Wang, Andriy Kovalenko, David Wishart, Neil R. Cashman

**Affiliations:** 1 Department of Medicine, University of British Columbia, Vancouver, British Columbia, Canada; 2 University of British Columbia, Djavad Mowafaghian Centre for Brain Health, Vancouver, British Columbia, Canada; 3 Department of Mechanical Engineering, Edmonton, Alberta, Canada; 4 National Research Council of Canada, Edmonton, Alberta, Canada; 5 Department of Biological Sciences, University of Alberta, Edmonton, Alberta, Canada; 6 Department of Computing Science, University of Alberta, Edmonton, Alberta, Canada; INRA Centre de Jouy-en-Josas, FRANCE

## Abstract

Oligomeric amyloid *β* (A*β*) is currently considered the most neurotoxic form of the A*β* peptide implicated in Alzheimer’s disease (AD). The molecular structures of the oligomers have remained mostly unknown due to their transient nature. As a result, the molecular mechanisms of interactions between conformation-specific antibodies and their A*β* oligomer (A*β*O) cognates are not well understood. A monoclonal conformation-specific antibody, m5E3, was raised against a structural epitope of A*β* oligomers. m5E3 binds to A*β*Os with high affinity, but not to A*β* monomers or fibrils. In this study, a computational model of the variable fragment (Fv) of the m5E3 antibody (Fv5E3) is introduced. We further employ docking and molecular dynamics simulations to determine the molecular details of the antibody-oligomer interactions, and to classify the A*β*Os as Fv5E3-positives and negatives, and to provide a rationale for the low affinity of Fv5E3 for fibrils. This information will help us to perform site-directed mutagenesis on the m5E3 antibody to improve its specificity and affinity toward oligomeric A*β* species. We also provide evidence for the possible capability of the m5E3 antibody to disaggregate A*β*Os and to fragment protofilaments.

## Introduction

The most common form of dementia is associated with Alzheimer’s disease (AD), which is a fatal neurodegenerative disorder [1]. Typically, abundant presence of neurofibrillary tangles and senile amyloid plaques are displayed in individuals with AD [2]. The amyloid plaques predominantly composed of densely packed A*β* fibrils [3]. A*β* is the cleavage product of the transmembrane amyloid precursor protein by *β*- and *γ*-secretases. The chain length of A*β* varies depending on the cleavage site of *γ*-secretase [4]. The two most common forms of A*β* present in AD brain are A*β*1-40 (A*β*40) and A*β*1-42 (A*β*42). A*β*40 is the most abundant isoform overall, but A*β*42 is the dominant isoform in plaques [5].

Monomeric A*β* is amyloidogenic. A few A*β* monomers can aggregate to form what is called an oligomer. These oligomers can further nucleate the formation of higher order oligomers or fibrils. The correlation between the deposition of amyloid plaques and AD is not as strong as was initially thought [6]. Multiple immunotherapeutic efforts against A*β* fibrils has shown limited efficacy [7]. The monomeric form of A*β* has been shown to have physiological roles [8, 9], and thus should not be the target of a therapeutic approach against AD. A vaccination against the monomeric form of A*β* also induces an autoimmune response [10] therefore; the monomeric form should not be targeted by an antibody [11]. Recent studies have focused on A*β*Os as they are linked to the age of onset of AD [12], are more toxic than fibrils [13], and lead to cognitive impairment [14]. An oligomer-specific antibody may not have the disadvantages of antibodies against the fibrils and monomers.

In an attempt to discover the toxic A*β*Os responsible for AD, various A*β*Os-dimers [15], trimers [16], and dodecamers [16, 17] have been purified from diseased brains. Various protocols were also developed for generating synthetic A*β*Os, including A*β*-derived diffusible ligands [18], globulomers [19], amylospheroid [20], annular protofibrils [21], and toxic soluble Abeta assembly (TAbeta) [22]. A powerful approach for discovering the agent that causes AD is to raise oligomer-specific antibodies that can recognize only the toxic oligomeric form of A*β* and not its monomeric or fibrillar form [23, 24]. A monoclonal antibody that specifically recognizes toxic A*β*Os could be useful for neutralizing the toxicity of such oligomers. An oligomer-specific antibody could also be useful as a biomarker to distinguish AD from other dementing syndromes. Its cognate mimotope can also be used to immunize a patient to harness the host immune system [25].

The amino acid sequence of A*β* is identical in monomeric, oligomeric or fibrillar forms. An A*β* oligomer-specific antibody must therefore differentiate between the conformations of oligomers, and other forms of A*β*. The mouse monoclonal oligomer-specific antibody, m5E3, was raised against the cyclic CGSNKGC peptide (cSNK), the central five residues of which are native to the A*β* peptide, flanked by non-native cysteines to cyclize the immunogen. The residues 25GSNKG29 of A*β* can adopt a sharp turn conformation in some A*β*Os [26]. The K28 residue was hypothesized to be solvent-exposed in some A*β*Os [23, 26]. K28 on the contrary is known to typically form an internal salt bridge in A*β* fibrils [27–30]. The sharp turn at these residues and the solvent exposed K28 were assumed to differentiate the structure of A*β*Os from monomers and fibrils.

A*β* monomers need to adopt a sharp turn conformation at the epitope residues 25GSNKG29 in order to be recognized by m5E3. However, A*β* monomers are relatively disordered [31], and are unlikely to adopt this turn. Multiple m5E3 epitopes are usually located close to each other in fibrils preventing the individual epitopes to enter the binding pocket of m5E3.

The difficulty of isolating A*β*Os with a specific structure and presence of various A*β*Os with heterogeneous structures are among the main reasons behind the failures in developing therapeutics for AD [32]. Atomic-level resolution of A*β*O structures have proven elusive, perhaps due to the transience and plasticity of these entities, theoretical and experimentally-informed structural models have been proposed ranging from dimers to large aggregates characterized by different secondary and tertiary structures. The interactions of the model of m5E3 with published A*β*O models may provide a ranking of how likely they are to exist in vivo. We selected representative structures of A*β*Os to parse the ranking of reactivity. While it is possible that m5E3 is reactive with only subclasses of A*β*Os, it is also possible that the activity of m5E3 with these models will help validate a particular structure for plausibility. With experimental limitations to resolve tertiary structure of A*β*Os, oligomers have usually been reported by their sizes and secondary structures. A brief overview of the models of A*β*Os used in this work follows. A trimer resolved experimentally by Kreutzer et al. (Panel A of S1 Fig of Supporting Information (SI)) is made of *β*-hairpins in a triangular shape [33]. A tetramer was revealed experimentally by Streltsov et al. (Panel B of S1 Fig of SI) with individual A*β* peptides forming two connected loop conformations [34]. An octadecamer developed based on experimental constraints by Gu et al. (Panel C of S1 Fig of SI) is made of stacks of *β*-sheets from individual A*β* peptides with three antiparallel *β*-strands [35]. A hexamer proposed theoretically by Shafrir et al. (Panel D of S1 Fig of SI) has a *β*-barrel structure with individual peptides forming three antiparallel *β*-strands [36]. A hexamer hypothesized theoretically by Laganowsky et al. (Panel E of S1 Fig of SI) has a nanotube-like conformation with individual *β*-strands [37]. A dodecamer assembled theoretically by Gallion (Panel F of S1 Fig of SI) is composed of two stacked disc-shaped sub-units. The discs are built of A*β* peptides from the tetramer by Streltsov et al., and have *α*-helical N-terminal residues [38]. It is worth noting that a wide range of structural features for proposed molecular models of A*β*Os may be indicative of a polymorphic nature for oligomers.

We hereby characterize different structural features of the above models relevant in the context of oligomer recognition by the m5E3 antibody. K28 is solvent-exposed in the trimer by Kreutzer et al., some chains of the tetramer by Streltsov et al., the last layer of *β*-sheets of the octadecamer by Gu et al., the hexamer by Shafrir et al., the hexamer by Laganowsky et al., and partly in some chains of the dodecamer by Gallion. A sharp turn at the epitope residues is formed in the trimer by Kreutzer et al., the hexamer by Shafrir et al., and the octadecamer by Gu et al. A wide-turn at the epitope residues is formed in the tetramer by Streltsov et al. and the dodecamer by Gallion. In the hexamer by Laganowsky et al. the epitope residues do not form a turn structure.

As A*β* fibrils are stable, various experimental structures are available for them. The structure of a fibril with three-fold symmetry was revealed by Lu et al. (Panel A of S2 Fig of SI) with a wide-turn at the epitope residues and a salt bridge between K28 and D23 of the same chain [27]. A structure of a fibril with two-fold symmetry was resolved by Petkova et al. (Panel B of S2 Fig of SI) with a wide-turn at the epitope residues and a salt bridge between K28 and D23 of either ±2 neighboring strands [28]. Schmidt et al. determined the structure of a dimer with a zipper-like two-fold symmetry in a fibril (Panel C of S2 Fig of SI) with no turn conformation at the epitope residues and a partially solvent-exposed K28 [29].

A model for a cross-*β* sub-unit was reported by Lührs et al. based on the observed protofilament of a fibril (Panel D of S2 Fig of SI) with a wide-turn at the epitope residues and a salt bridge between K28 and D23 of the adjacent chains [39]. Xiao et al. presented a model for a cross-*β* sub-unit (Panel E of S2 Fig of SI) with no sharp turn in the epitope region. This model’s partly solvent-exposed K28 forms a salt bridge with the carboxyl of C-terminus A42 of the same chain and not D23 [40]. The structure of the synthetic A*β* fibrils containing such A*β* cross-*β* sub-units was also published by both Colvin et al. [41] and Wälti et al. [30] (Panel F of S2 Fig of SI). Various features of the oligomers, fibrils and cross-*β* sub-units are summarized in S1 Table of SI.

An established method of classifying A*β*Os experimentally is based on conformation-specific antibodies [42]. Here, we computationally classify the models of A*β*Os as Fv5E3-positives or Fv5E3-negatives using our Fv model of the m5E3 antibody. Understanding how m5E3 binds to its A*β* cognates can be used to design even better monoclonal or single chain variable fragment (ScFv) oligomer-specific antibodies. In the following sections, we first present an Fv model for the m5E3 antibody and show how it detects its cyclic mimotope. We then explore the molecular mechanisms of Fv5E3 interaction with A*β*Os. Finally, we show why m5E3 has a lower affinity for A*β* fibrils. We distinguish between the fibrils and the cross-*β* sub-units and explain how Fv5E3 interacts with the cross-*β* sub-units.

## Results

### A variable fragment (Fv) model of the m5E3 conformation-specific monoclonal antibody

The sequence of the A*β*42 monomer is DAEFRHDSGYEVHHQKLVFFAEDV*GSNKG* AIIGLMVGGVVIA. The residues 25-29 of A*β*42 (GSNKG) with a solvent-exposed lysine in a sharp turn conformation were hypothesized to be the epitope of an A*β*O specific antibody [23]. The residue K5 is solvent-exposed when the epitope residues are cyclized by a disulfide bond, CGSNKGC (cSNK). The disulfide bond also forces the turn structure of GSNKG to be sharp. The mouse monoclonal antibody 5E3 was raised against cSNK. It has been demonstrated that the m5E3 antibody has a much higher affinity for A*β*Os compared to A*β* fibrils or monomers [23].

**Fig 1:**
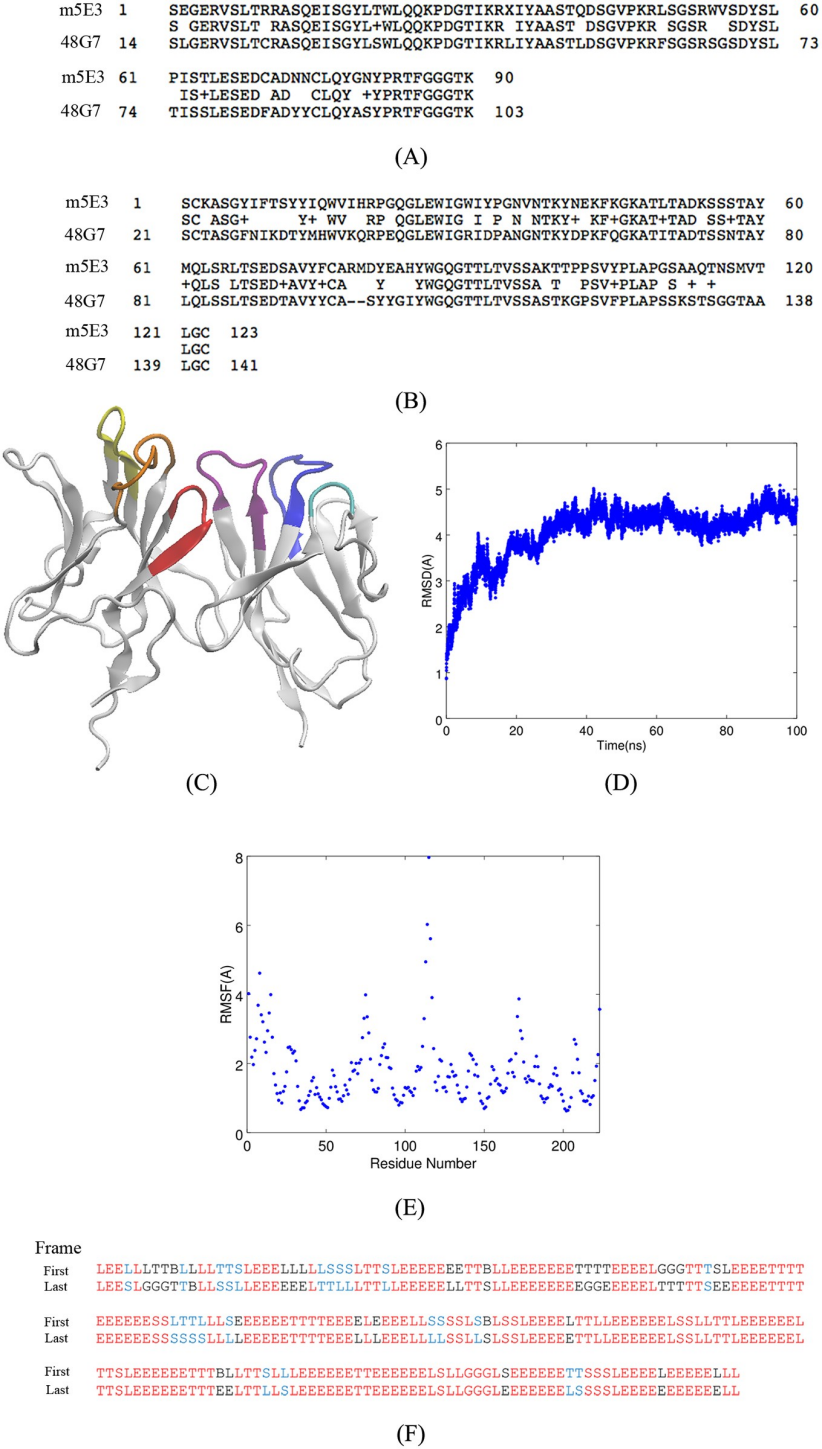
**A**) Alignment of the m5E3 and 48G7 light chains. **B**) Alignment of the m5E3 and 48G7 heavy chains. **C**) Model of the m5E3 antibody built from the framework of the 48G7 Fab fragment. Complementarity determining region 1 (CDR1), CDR2, and CDR3 of the light chain are shown in blue, cyan and purple colors, respectively. CDR1, CDR2, and CDR3 of the heavy chain are shown in orange, yellow and red colors, respectively. **D**) LRMSD of C_*α*_ atoms of Fv5E3 during a 100 *ns*-long MD simulation. **E**) The root mean square fluctuation (RMSF) of C_*α*_ atoms of Fv5E3 during the 100 *ns*-long MD simulation. The heavy chain is from residue 1 to 115 and the light chain is from 116 to 223. **F**) The secondary structure content of Fv5E3 from the first and last frames of the 100 *ns*-long MD simulation. The letters B and E stand for isolated *β*-bridge and extended *β*-sheet, respectively. The letter G stands for 3_10_ helix. The letters T, S, and L stand for hydrogen bonded turn, bend, and unstructured loop, respectively.

Here, we present a model for the Fv fragment of the m5E3 antibody. We used this model to study how m5E3 binds to A*β*Os, and why it has a low affinity for A*β* fibrils. After translating the partial nucleotides’ sequence of m5E3 [43] to the corresponding amino acid sequence using the online ExPASy server (https://web.expasy.org/translate/) [44], we obtained the partial sequences for the light and heavy chains of m5E3. A search for a similar framework for m5E3 using NCBI’s BLAST tool (blastp algorithm) with default parameters (https://blast.ncbi.nlm.nih.gov/Blast.cgi) returned the Fab (fragment, antigen binding) 48G7 (pdb entry 2rcs) [45]. The BLAST score for the light and heavy chains were 147 and 151, respectively. The corresponding E-values were 5e-43 and 2e-51. These alignments indicate that there is an 83% identity between the light chains of m5E3 and 48G7, and a 63% identity between the heavy chains of m5E3 and 48G7 (Panels A and B of Fig 1).

To build a homology Fv model for m5E3, we mutated the residues of the Fv region of 48G7 Fab fragment to the corresponding residues of the m5E3 antibody. We also used the Antibody module of Rosetta software to predict an Fv model for m5E3 [46]. The models obtained with these two approaches are very similar. Their least root mean square deviation (LRMSD) of C_*α*_ atoms is only 3.6 Å. In this paper, we use the homology model of m5E3 built from the framework of 48G7. The docking algorithm [47] takes advantage of the same components of the homology modeler [46] used to build a structural model of the antibody, and remodels the antibody in presence of each antigen. The CDRs of the m5E3 antibody were determined using the protocol provided in Ref. [48]. CDR1, CDR2, and CDR3 of the light chain of m5E3 include residues RASQEISGYLT, AASTQDS, and LQYGNYPRT, respectively. CDR1, CDR2, and CDR3 of the heavy chain of m5E3 include residues ASGYIFTSYY, IYPGNVNT, and ARMDYEAHY, respectively. The resulting Fv model of m5E3 with the highlighted CDRs is shown in Panel C of Fig 1. The model is stable as assessed in a 100 *ns*-long MD simulation. The LRMSD of this model, with over 200 C_*α*_ atoms, only changes by about 5 Å during the simulation (Panel D of Fig 1). The RMSF of the C_*α*_ atoms of Fv5E3 also demonstrate that the main fluctuations occur at the N-termini and C-termini residues of the heavy and light chains. The residues 74-76 of the heavy chain which are part of a non-CDR turn conformation, and the residues following the CDR2 of the light chain are also flexible. (Panel E of Fig 1). The secondary structure content of the model does not vary much during the simulation as determined by the Wordom software [49] (Panel F of Fig 1). The relaxed Fv5E3 obtained in this simulation was used in docking simulations.

Throughout this paper, we use the term m5E3 for the monoclonal 5E3 antibody to refer to experimental interactions. The term Fv5E3 is reserved for the computational Fv model of m5E3 if we refer to interactions in silico.

### Interaction between Fv5E3 and the cyclic mimotope of m5E3

**Fig 2:**
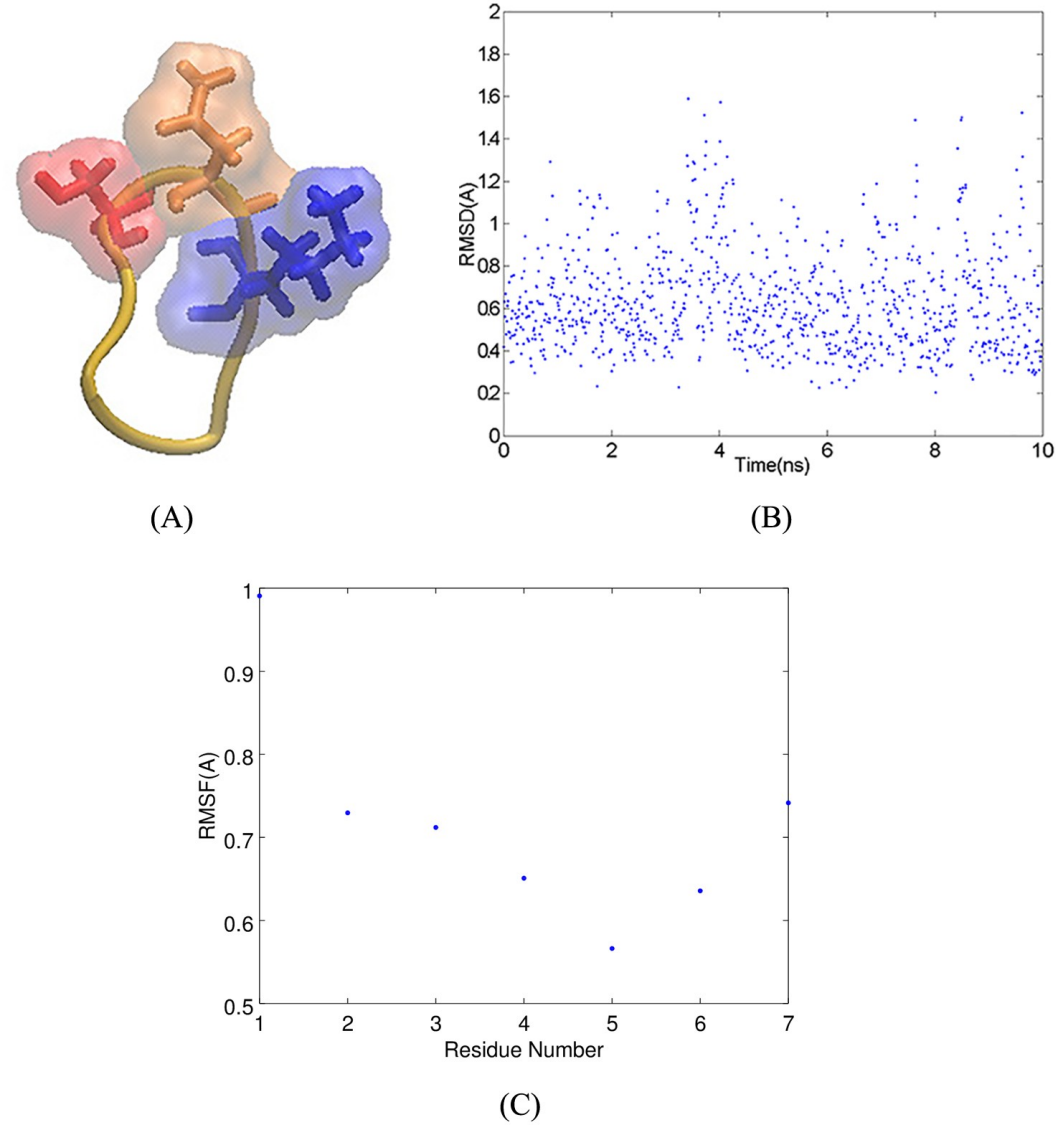
**A**) Cyclic CGSNKGC mimotope. The SNK residues are shown in stick and solvent-exposed surface representations in red, orange and blue, respectively. **B**) The LRMSD of heavy atoms of cSNK in a 10-*ns* MD simulation. **C**) The RMSF of C_*α*_ atoms of cSNK in the 10-*ns* MD simulation.

In this section, we analyze how the cSNK mimotope interacts with Fv5E3 [50]. The molecular model of cSNK was obtained in a previous study (Panel A of Fig 2). The LRMSD of this model from a 10-*ns* MD simulation performed in the current study confirms that the model represents an average conformation of the cSNK peptide (Panel B of Fig 2). The backbone atoms of cSNK are restrained due to the presence of the disulfide bond (Panel C of Fig 2). The cSNK residues are therefore forced to stay within a certain distance from each other. The distance between the C_*α*_ atoms of G2 and G6 of cSNK is 5.55 Å. There is no space within this sharp turn for K5 to be buried; therefore, the side chain of K5 is always solvent-exposed. We use the distance between the C_*α*_ atoms of G2 and G6 as a measure of how sharp the turn conformation in the models of A*β* aggregates is.

Fv5E3 has three solvent-exposed acidic residues in its CDRs. These residues are E28 in CDR1 of the light chain, and D100 along with E102 from CDR3 of the heavy chain. The cSNK has a net charge of +1 at a neutral pH. The negative charges of the acidic CDR residues located at the solvent-exposed surface of Fv5E3 create an affinity for the positive charge of the K5 residue of cSNK. The presence of a strong negative electrostatic field around the acidic residue E102 in CDR3 of the heavy chain was determined using the APBS electrostatics plugin of the VMD software [51] (S3 Fig of SI). We believe that this region is the main binding pocket for cSNK, and that the initial detection of cSNK by the m5E3 antibody is driven mainly electrostatically.

**Fig 3:**
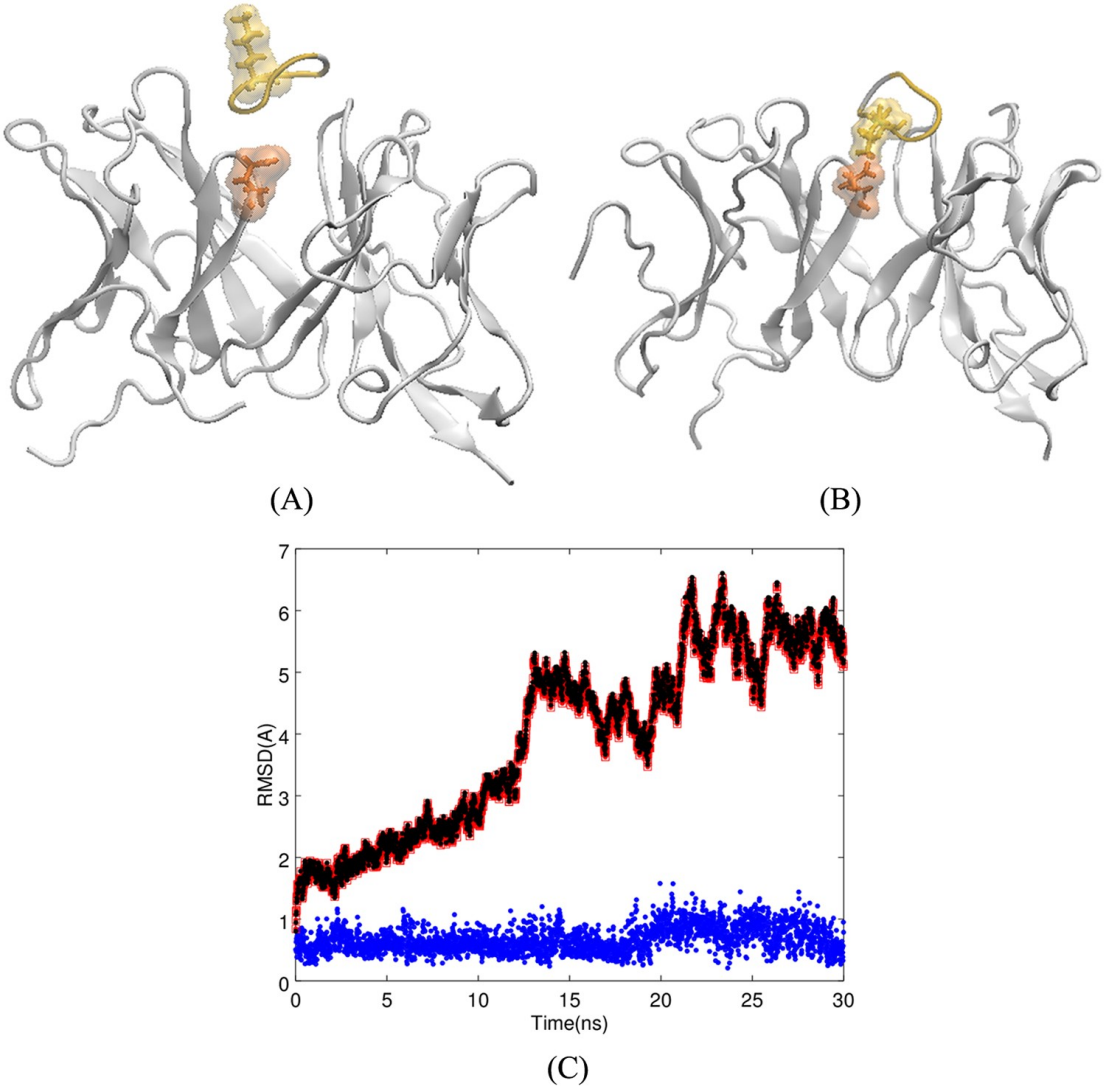
**A**) Top-ranked docked structure of Fv5E3 and cSNK. K5 residue of cSNK and the E102 residue of Fv5E3 are shown in solvent-exposed surface and stick representations in yellow and orange, respectively. **B**) Complex after 30 *ns* of MD simulation. **C**) The LRMSD of C_*α*_ atoms of the complex (red), Fv5E3 (black), and cSNK (blue) during the 30-*ns* MD simulation.

To further elucidate how cSNK interacts with m5E3, we first docked cSNK to our Fv model of the m5E3 antibody. In the top hundred complexes from the docking simulation, the cSNKs are docked mainly in the superior gap (based on the orientation of the model in Panel C of Fig 1) between the light and heavy chains. The top-ranked docked structure of Fv5E3 and cSNK with the Rosetta score of -150.59 (-21.51 per residue) provides an initial complex to study how cSNK interacts with Fv5E3 (Panel A of Fig 3). The cSNK peptide does not deviate substantially from its initial docked binding site during a 30-*ns* simulation (Panels A and B of Fig 3). There is a small 3 Å increase in LRMSD of the complex in the first 15 *ns*, which could be because of the adjustments by the antibody for the presence of cSNK (Panel C of Fig 3). The simulation converges towards the last 10 *ns*. The small 2-3 Å fluctuations in the last 15 *ns* are due to the changes by the antibody to adjust for the small fluctuation of the cyclic peptide in the binding pocket (Panel C of Fig 3 in black).

The backbone of G6 from the cSNK peptide forms high occupancy hydrogen bonds with D100 of the heavy chain, and G92 of the light chain of Fv5E3. G6 also forms low occupancy hydrogen bonds with the residues Y33 of the heavy chain, Y94 and R96 of the light chain of Fv5E3 (S2 Table of SI). Hydrogen bonds formed by G6 make it an anchor to keep cSNK in the binding pocket. A salt bridge (a hydrogen bond accompanied by an ionic interaction) is formed between the E102 of CDR3’s heavy chain, and K5 of cSNK with high occupancy (Panel B of Fig 3, S2 and S3 Tables of SI). The K5 of cSNK also forms hydrogen bonds with the residues M99, and D100 from CDR3 of the heavy chain (S2 Table of SI). K5 acts as an additional anchor to stabilize the complex. There is also a cation-*π* interaction between K5 of cSNK and Y32 of CDR1 of the heavy chain of Fv5E3 (S3 Table of SI). The other (low occupancy) hydrogen bonds are formed between the G2 and S3 of cSNK, and the G92 and Y32 residues of Fv5E3, respectively. No hydrogen bonds are formed between N4 of cSNK, and the antibody. No hydrophobic interaction was identified between the final conformation of Fv5E3 and cSNK from the 30-*ns* simulation.

While in the binding pocket, cSNK does not dissociate from Fv5E3 even after its lysine (K5) is mutated to a glycine. This is supported by an MD simulation. Thus, the stability of the cSNK-Fv5E3 complex is due to the many hydrogen bond interactions formed between cSNK and the antibody. The average binding free energy for the association of cSNK and Fv5E3 in pure water during the simulation is -41.56 kcal/mol (standard deviation (std. dev.) of 4.97), which indicates that the interaction between cSNK and Fv5E3 is a favorable one. The K5 of cSNK as expected has a favorable pairwise contributions to the binding free energy from interactions with E102 (-10 kcal/mol), D100 (-6.15 kcal/mol), and with M99 (-4.96 kcal/mol), all from the heavy chain of the antibody. Contribution to the binding free energy from the interaction of G6 of cSNK with D100 of the heavy chain is -4.20 kcal/mol.

Previously, a similar computational approach was used to compare the stability of another cyclic peptide and its linear form for a different antibody [52]. As a negative control, we carried out docking simulation of cSNK, and the B10 fibril specific antibody fragment [53]. In the top hundred docked complexes, cSNK interacts predominantly to the framework of B10, and only rarely close to the CDRs of B10.

### Interaction between Fv5E3 and A*β* oligomers

In the following sections, we go through the models of A*β*Os proposed in the literature and classify them into Fv5E3-positives and possibly Fv5E3-negatives. This classification was performed based on the combination of docking and MD simulation results. We believe Fv5E3-positive A*β* aggregates should have structural characteristics similar to cSNK that is a sharp turn at the epitope residues G25-G29, a solvent exposed K28, and available space around a few of the turns to enter the binding pocket of Fv5E3. In the following sections, we also reveal the molecular details of the interactions between Fv5E3 and the cognate A*β*Os for which structures are either resolved experimentally or predicted computationally.

#### Experimental models of A*β* oligomers

A*β*Os are transient entities; this has made it difficult to determine their molecular structures. To overcome the transient nature of A*β*Os, various modifications have been performed on the sequence of A*β* to generate stabilized oligomers. It is hard to judge whether these modified constructs represent well the structure of the physiologically relevant A*β*Os. Below, we analyze how these proposed experimental models of A*β*Os interact with Fv5E3.

#### The trimer model of A*β*17-36 oligomers by Kreutzer et al

The crystal structure of a trimer from a cyclized A*β*17-36 was determined by Kreutzer et al. [33] (pdb entry 5hoy, Panel A of S1 Fig of SI). Higher order oligomers were observed to form from these trimers, as each trimer has two large hydrophobic surfaces [33]. Since the trimers are the building block of these higher order oligomers, we focus on the interaction between Fv5E3 and an individual trimer. To force the formation of a *β*-hairpin by the A*β*17-36 peptide, an extra ornithine residue was introduced at position 16 of each individual peptide. The amino group of the side chain of this ornithine residue is connected to V36. The residues V24 and G29 were also mutated to cystines. The disulfide bond between the cystines stabilizes the *β*-hairpin conformation. The residue G33 was also N-methylated (sarcosine) to avoid uncontrolled aggregation in vivo. We used a disulfide bond instead of the ornithine bond for the docking and MD simulations. We also used a glycine instead of the sarcosine at position 33 in silico. The G25-G29 residues form a sharp turn in the trimer model by Kreutzer et al. with a distance of 6.6 Å from G25 to G29, and there is plenty of space between the turns to allow a turn to enter the binding pocket of Fv5E3. The K28 residues are also solvent exposed in this model.

**Fig 4:**
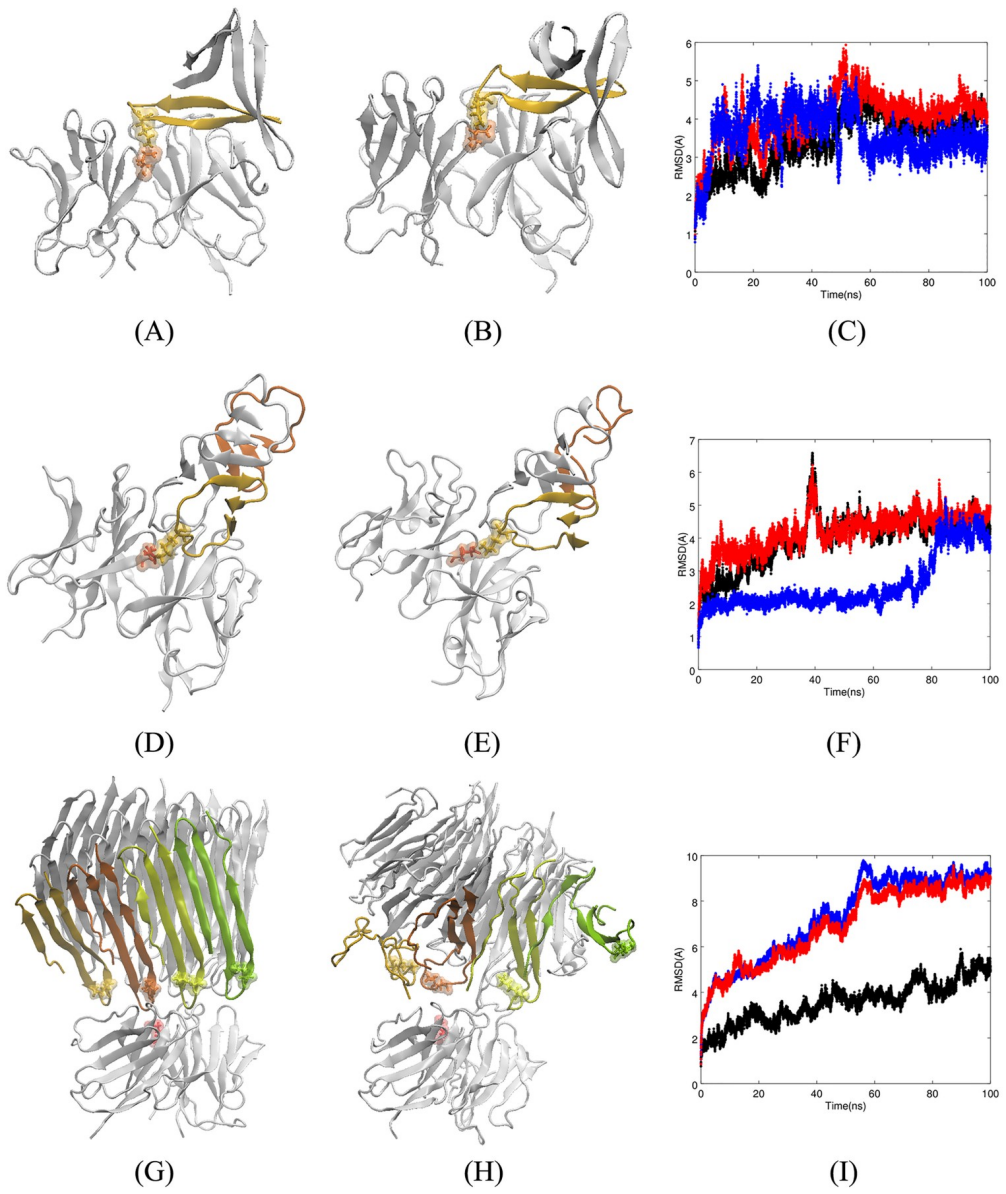
**A**) Top-ranked docked structure of Fv5E3 and the trimer by Kreutzer et al. The stick and solvent-exposed surface representations of the K28 residue of chain B of the trimer and the E102 of the heavy chain of Fv5E3 are shown in yellow and orange colors, respectively. **B**) Complex after 100 *ns* of MD simulation. **C**) The LRMSD of C_*α*_ atoms of the complex (red), Fv5E3 (black), and the oligomer (blue) during the 100-*ns* MD simulation. **D**) Top-ranked docked structure of Fv5E3 and the tetramer model of A*β*Os by Streltsov et al. The stick and solvent-exposed surface representations of the K28 residue of the chain D of the tetramer, and the E102 residue of the heavy chain of Fv5E3 are shown in yellow and red, respectively. Chain E is shown in orange. **E**) Complex after 100 *ns* of MD simulation. **F**) The LRMSD of C_*α*_ atoms of the complex (red), Fv5E3 (black), and the tetramer (blue) during the 100 *ns* MD simulation. **G**) Top-ranked docked structure of Fv5E3 and the octadecamer by Gu et al. The chains that are close to Fv5E3 are shown in various colors. **H**) Complex after 100 *ns* of MD simulation. **I**) The LRMSD of C_*α*_ atoms of the complex (red), Fv5E3 (black), and the prefibrillar oligomer (blue) during the 100 *ns* MD simulation.

To show how Fv5E3 interacts with the trimer by Kreutzer et al., we performed a docking simulation. Fv5E3 interacts with the turn conformation at the epitope residues, the edge of the *β*-strands, or rarely with the two hydrophobic surfaces within the three *β*-hairpins, in the top hundred docked complexes. Fv5E3 interacts with the turn conformation at the epitope residues of the trimer by Kreutzer et al. in the top-ranked docked structure with the Rosetta score of -206.45 (-3.27 per residue, Panel A of Fig 4). The three-dimensional structures of the individual A*β* peptides are fairly preserved during the 100-*ns* MD simulation (Panel B of Fig 4). The individual C_*α*_ atoms fluctuate substantially as it is apparent from the broadened LRMSD line (Panel C of Fig 4 in blue). The quaternary triangular structure is also lost. Despite the loss of the triangular shape, the trimer stays as a trimer (Panel B of Fig 4) and does not disaggregate as the overall LRMSD is plateaued during the simulation (Panel C of Fig 4 in blue). It would be interesting to see if two or three Fv5E3 simultaneously bound to the trimer can disaggregate it. The simulation converges in the last 50 *ns* (Panel C of Fig 4 in red). The change in the LRMSD of the complex in the first 50 *ns* of the simulation is mostly because of the minor adjustments by the antibody to account for the presence of the oligomer (Panel C of Fig 4 in black).

While some of the hydrogen bonds between Fv5E3 and the trimer are formed with the framework residues of Fv5E3 (S4 Table of SI), a salt bridge with high occupancy is formed between the K28 of the oligomer and the E102 of the heavy chain of Fv5E3 (S4 and S5 Tables of SI). The hydrophobic and ionic interactions may also partially stabilize the interaction between Fv5E3 and the trimer (S5 Table of SI). There are no aromatic-aromatic, aromatic-sulphur or cation-*π* interactions formed between them. The average binding free energy for the association of the oligomer and Fv5E3 during the simulation is -31 kcal/mol (std. dev. of 7.43). This negative average binding free energy is indicative of a favorable interaction between the oligomer and the antibody. The most favorable interactions contributing to the binding free energy are between E22 of chain A of the trimer and K60 (-13.45 kcal/mol), S56 (-4.8 kcal/mol) of the light chain, K28 of chain B of the trimer and E102 (-4.23 kcal/mol) of the heavy chain, and G33 of chain B of the trimer and Q54 of the light chain (-2.94 kcal/mol). The sum of pairwise contributions to the binding free energy involving the hydrophobic residues that form the two hydrophobic surfaces of the trimer is not a large positive number (-0.31 kcal/mol) which may indicate that the trimer will not dissociate from the antibody.

#### The tetramer model of A*β*18-41 oligomers by Streltsov et al

A crystal structure for a tetrameric A*β*18-41 oligomer was resolved by Streltsov et al. [34] (pdb entry 3moq, Panel B of S1 Fig of SI). Each A*β* molecule was stabilized by a chimerical fusion with a shark immunoglobulin new antigen receptor. The residues G25-I31 form a solvent-exposed wide-turn structure with a distance of 11.66 Å between G25 and G29. The side chains of K28s in two of the chains (A/D) are solvent-exposed, and do not form any hydrogen bond. The antibody m5E3 may be able to detect an individual tetramer as the K28 residues are part of turns, and do not always form salt bridges. There is also enough space around the epitope turn to allow its entrance into the binding pocket of m5E3. The top and bottom of the tetramers are covered by hydrophobic surfaces. The hydrophobic residues are also running alongside the tetramer. The tetramer is therefore hypothesized as the building block of higher order oligomers. It seems unlikely that m5E3 would be able to detect multimer oligomers made from these tetramers, as their close packing may not allow an individual epitope to enter the binding pocket of m5E3.

The docking simulation also predicts a possible interaction between the tetramer and Fv5E3. The top hundred docked complexes show a similar mode of interaction between Fv5E3 and the tetramer as in the top-ranked complex with a negative score of -247.37 (-2.51 per residue, Panel D of Fig 4). The tetramer after 100 *ns* of MD simulation stays bound to Fv5E3 (Panel E of Fig 4). One of the two chains of the tetramer (chain E) that is not interacting with Fv5E3 starts to unfold (Panels D and E of Fig 4 in orange). The reason for the 1.5 Å increase of the oligomer’s LRMSD during the last 20 *ns* of the simulation is also the unfolding of chain E of the oligomer (Panel F of Fig 4 in blue). A longer simulation may demonstrate if the oligomer disaggregates at some point. The A*β*18-41 within the fusion complex and the A*β*17-42 variant of the tetramer by Streltsov et al. were shown to be stable in simulations [54]. The complex and the antibody are fairly stable during the simulation (Panel F of Fig 4 in red, and black, respectively), and the simulation converges in the last 20 *ns*. The small 2-3 Å fluctuation in the LRMSD of the complex and the antibody about 40 *ns* in the simulation is due to the fluctuation of the N-termini residues of the Fv fragment.

The two epitope residues that form high occupancy hydrogen bonds with Fv5E3 are N27 and K28 (S4 Table of SI). Specifically, the N27 residue of chain B of the oligomer forms hydrogen bonds with D100, E102 and Y32 of the heavy chain of Fv5E3 with high occupancies. The K28 residue of chain D of the oligomer forms a salt bridge with E102 of the heavy chain of Fv5E3 (S4 and S5 Tables of SI). Hydrophobic interactions also play an important role in stabilizing the binding of Fv5E3 to the tetramer (S5 Table of SI). The residue V24, which precedes the epitope residue G25, participates in a hydrophobic interaction with A50 of CDR2 of Fv5E3’s light chain with a fairly high occupancy. There are also aromatic-aromatic interactions between Fv5E3 and the oligomer (S5 Table of SI). No aromatic-sulphur or cation-*π* interactions exist at the end of the simulation between Fv5E3 and the tetramer.

The interaction between the tetramer of Streltsov et al. and Fv5E3 is favorable as its average binding free energy is negative (-17.10 kcal/mol with std. dev. of 3.26). We note however that residues 1-17 are not present in this model, and their presence may block Fv5E3’s access to the epitope residues G25-G29. The interaction between K28 of chain D of the tetramer and E102 of the heavy chain of the antibody contribute most to the binding free energy (-16.04 kcal/mol). The interactions between N27 of chain B of the tetramer and D100 (-5.29 kcal/mol), E102 (-3.88 kcal/mol) and Y32 (-3.15 kcal/mol) of the heavy chain of Fv5E3 are the next best contributors to the binding free energy.

#### The octadecamer model of A*β* oligomers by Gu et al

An octadecamer model of A*β*Os based on restraints from the site-directed spin labeling and electron paramagnetic resonance studies was presented by Gu et al. [35]. Gu et al. stabilized A*β*Os by fusing the sequence of A*β*42 with the sequence of the chaperone GroES followed by the sequence of the ubiquitin protein. With the latter fusion, this aggregate is trapped in the oligomeric state with fibril-like *β*-sheets (Panel C of S1 Fig of SI).

The residues G25-G29 form a sharp turn in this model with a distance of 7.8 Å between G25 and G29. As the epitope turn is sharp, the side chain of K28 residue is not buried inside the turn, and does not form a salt bridge with D23 of its own strand. It is however trapped between adjacent *β*-sheets, and can form a salt bridge with D23 of the adjacent *β*-strand in the next *β*-sheet. The distance between the amine of K28 and carboxyl of D23 in this model is 1.88-5.57 Å. When the K28 residue does not form a salt bridge with D23, it participates in an ionic interaction with the mainchain carbonyl oxygen of V24 of mostly the same chain. It can also participate in an ionic interaction with the mainchain carbonyl oxygen of K28 in an adjacent *β*-sheet. In the last layer of *β*-sheets, the K28 residues are solvent-exposed (Panel C of S1 Fig of SI in red), where they may form an intrachain salt bridge with E22. Since the side chains of K28 residues are fully engaged in hydrogen bonding, salt bridges and ionic interactions within the octadecamer model of A*β*Os, and there is little space around each epitope turn to allow the entrance of a small number of epitopes to the binding pocket of the m5E3 antibody, we anticipate that this prefibrillar oligomer cannot be detected by the m5E3 antibody specifically through the epitope residues.

Contrary to our prediction, the result of the docking simulation shows a possible interaction between this model of A*β*Os and the Fv model of the antibody close to the epitope residues. In the top hundred docked complexes, Fv5E3 binds along the epitope turns of the octadecamer. This interaction for the best docked complex with the Rosetta score of -596.59 (-0.88 per residue) is shown in Panel G of Fig 4. Despite a favorable Rosetta score, the complex is not stable in a 100-*ns* simulation (Panel I of Fig 4 in red). The layer of *β*-sheets in the octadecamer model close to Fv5E3 is disrupted during the simulation (Panel H of Fig 4). The tertiary structure of the two layers of *β*-sheets in the middle are fairly preserved. The C-termini *β*-hairpin of one of the A*β* peptides in the layer of *β*-sheets furthest from Fv5E3 also moves away from the rest of the octadecamer. As the octadecamer goes through a lot of changes, Fv5E3 makes adjustments to try to detect it (Panel I of Fig 4). With many changes in the layer close to Fv5E3, it seems plausible to hypothesize that Fv5E3 is responsible for this disaggregation. A simulation of the octadecamer model by itself should be performed in a follow-up study to confirm this hypothesis.

In the course of the MD simulation, the occupancies of the hydrogen bonds between the prefibrillar oligomer of Gu et al. and Fv5E3 are however low (S4 Table of SI). The occupancies of hydrophobic interactions are low as well (S5 Table of SI). There are also no salt bridges, aromatic-aromatic, aromatic-sulphur or cation-*π* interactions present between the final complex of Fv5E3 and the oligomer. As the LRMSDs do not converge, it is not possible to properly estimate the binding affinity between the oligomer and Fv5E3.

#### Computational and theoretical models of A*β* oligomers

In addition to the experimental models considered in the previous sections, some of the structural models of the oligomers have been built computationally based on available experimental constraints, and the rest of the proposed models are purely theoretical and do not rely on any experimental data. The computational and theoretical models may not be as accurate as the experimentally resolved models presented in the previous section. However, the analyses of their interactions with Fv5E3 allow us to propose experiments with oligomers that have similar structures. In the following sections, we will assess Fv5E3’s ability to bind to these computational and theoretical models of the oligomers.

#### The hexamer model of A*β*42 oligomers by Shafrir et al

Shafrir et al. developed many computational barrel-based models for soluble and membrane-bound A*β*Os [36]. The formation of *β*-barrels is a natural mechanism that keeps *β*-sheets from growing into larger aggregates [55]. Eleven of these models are for soluble hexameric A*β*Os. These models differ mainly in the secondary structures (*β*-sheets vs *α*-helices) of the three regions defined by Shafrir et al. for A*β* (D1-H14, Q15-K28, and G29-A42), and in their orientations with respect to each other (parallel vs antiparallel). It is not feasible to work on every single *β*-barrel model proposed by Shafrir et al., and such an inspection does not seem to provide us much more information regarding how *β*-barrel models might interact with Fv5E3 than the study of a single one. Here, we choose one of these models as a representative of the *β*-barrel models.

In this hexameric *β*-barrel model, each A*β* monomer is made of three antiparallel *β*-strands. Parallel *β*-strands of adjacent A*β* peptides align with each other around the model (Panel D of S1 Fig of SI). K28s are solvent-exposed in this model, and in six other *β*-barrel models proposed by Shafrir et al. In three of the models, the K28s’ side chains are located on the surface, but they do not stick out to the solvent, and are stabilized by interactions with other residues. In one of the eleven *β*-barrel models; however, K28s of three chains are completely buried inside the turns.

For the selected *β*-barrel model, sharp turns at the epitope residues are formed with a distance of 7.30 Å between G25 and G29 of each chain. In six *β*-barrel models, sharp turns are present at the epitope residues. The uncrowded space around the epitope residues of the selected *β*-barrel model allows these residues to enter the binding pocket of Fv5E3. In nine other *β*-barrel models by Shafrir et al., enough space around the epitope residues are also available to enter the binding pocket of Fv5E3.

**Fig 5:**
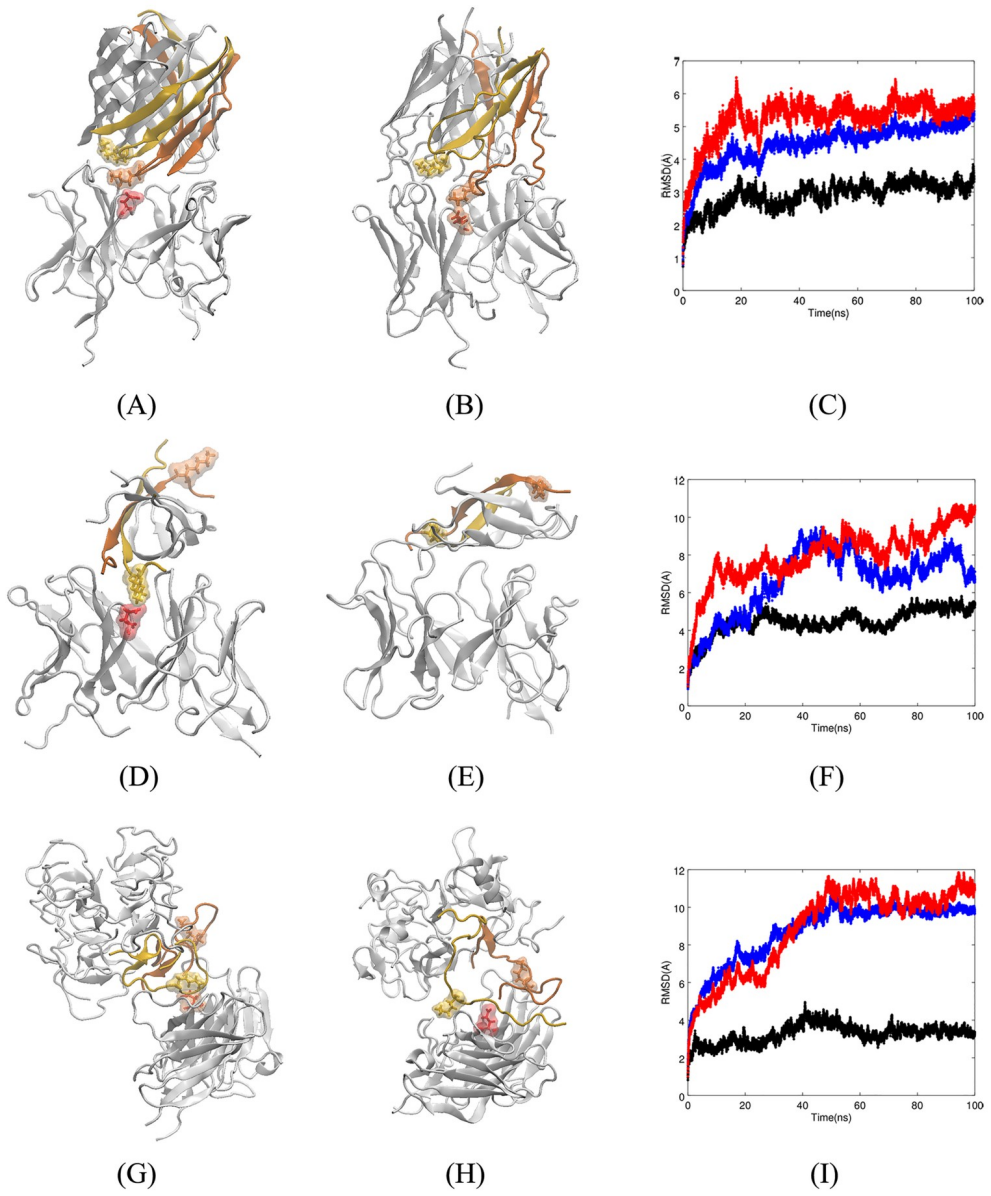
**A**) Top-ranked docked structure of Fv5E3 and the hexamer by Shafrir et al. Two K28 residues of the oligomer and the E102 residue of Fv5E3 are shown in solvent-exposed surface and stick representations in yellow, orange, and red, respectively. **B**) Complex after 100 *ns* of MD simulation. **C**) The LRMSD of C_*α*_ atoms of the complex (red), Fv5E3 (black), and the oligomer (blue) during the 100-*ns* MD simulation. **D**) Top-ranked docked structure of Fv5E3 and the hexamer by Laganowsky et al. The K28 residues of two chains of the oligomer, and the E102 residue of the heavy chain of Fv5E3 are shown in yellow, orange and red colors, respectively. **E**) Complex after 100 *ns* of MD simulation. **F**) The LRMSD of C_*α*_ atoms of the complex (red), Fv5E3 (black), and the oligomer (blue) during the 100-*ns* MD simulation. **G**) Top-ranked docked structure of Fv5E3 and the disc-shaped oligomers. The stick and surface representations of K28 residues of two of the chains of the oligomer and the E102 residue of Fv5E3 are shown in yellow, orange and red, respectively. **H**) Complex after 100 *ns* of MD simulation. **I**) The LRMSD of C_*α*_ atoms of the complex (red), Fv5E3 (black), the oligomer (blue) during the 100 *ns* MD simulation.

In the top hundred docked complexes, Fv5E3 binds to the edge around the top (based on the view of the model in Panel D of S1 Fig of SI) of the hexamer by Shafrir et al., where K28s’ side chains are sticking out to the solvent. Only in one of the top hundred complexes Fv5E3 interacts with the side of the barrel. Fv5E3 interacts with a GSNKG turn of the hexamer by Shafrir et al. in the best docked complex with the Rosetta score of -416.88 (-1.65 per residue, Panel A of Fig 5). The interaction between Fv5E3 and the hexamer is stable during the last 80 *ns* of the trajectory (Panel C of Fig 5 in red). The antibody is also fairly stable during the simulation with minor adjustments following the changes in the oligomer (Panel C of Fig 5 in black). The LRMSD of the oligomer however does not reach a plateau during the course of the simulation (Panel C of Fig 5 in blue). The hexamer by Shafrir et al. seems to start to disaggregate in the presence of Fv5E3 after 100 *ns* of MD simulation (Panel B of Fig 5). Involvement of the chains of the A*β*O in many interactions with Fv5E3 possibly will lead to the separation of those chains from the A*β*O. A longer simulation of the complex is needed to confirm the disaggregation of the A*β*O by Fv5E3. The hexamer by itself was shown to be stable [36].

In the context of the oligomer recognition by the antibody, it is important to note that K28 of the chain B of the oligomer forms a high occupancy salt bridge with the heavy chain’s E102 of Fv5E3 (S6 and S7 Tables of SI). We think that this interaction is the driving force that brings the antibody and the oligomer together. This interaction was also present between Fv5E3 and the cyclic mimotope. Another hydrogen bond with high occupancy is between D1 of chain B of the hexamer, and R46 of Fv5E3 light chain’s framework (S6 Table of SI). The other low occupancy hydrogen bonds may contribute to the stability of the complex as well (S6 Table of SI).

The two high occupancy hydrophobic interactions during the course of the simulation are between A30 of chain E and I31 of chain G of the hexamer, and Y52 of the heavy chain and Y94 of the light chain of Fv5E3, respectively (S7 Table of SI). The other low occupancy hydrophobic, ionic and cation-*π* interactions may also contribute to the overall stability of the complex. No aromatic-aromatic or aromatic-sulphur interaction exists between Fv5E3 and the hexamer in the final conformation after 100 *ns*. The MM-GBSA average binding free energy of the complex is -30.46 kcal/mol (std. dev. of 7.98). This negative binding free energy confirms the favorable interaction between the hexamer by Shafrir et al. and Fv5E3. The interaction that contributes most to the binding free energy is between K28 of chain B of the oligomer and E102 of the heavy chain of Fv5E3 (-13.85 kcal/mol).

Other *β*-barrel models of A*β*Os have been proposed by Pan et al. [26], Lendel et al. [56], and Nguyen et al. [57]. Pan et al. developed a protocol for the in vitro generation of the small stable A*β*40 oligomers. The schematic representation of their model based on the mass spectrometry data represents a tetrameric *β*-barrel structure. Each A*β*40 is expected to form a *β*-hairpin conformation with a solvent-exposed K28 [26]. These *β*-barrel tetrameric oligomers of Pan et al. can be used for experimental verification of m5E3’s ability to bind to the *β*-barrel oligomers. These oligomers can also be used to examine whether m5E3 can disaggregate the *β*-barrel oligomers in vitro. The other interesting hexameric *β*-barrel model was developed by Lendel et al. as a building block for a modified A*β*42 protofibril [56]. The constituting modified A*β*42 peptides have two mutations at positions 21 and 30 to cystines. The undeposited coordinate file of this model prevents computational analysis of the interaction between this *β*-barrel model and Fv5E3. Nevertheless, some conclusions regarding Fv5E3’s ability to bind to this hexamer can be obtained based on structural features discussed in Ref. [56]. The residues A24-N27 of this model form a sharp turn. Since this turn is sharp, the side chain of K28 cannot be buried inside the turn. The K28 side chain is solvent-exposed. The K28 residue however makes a salt bridge with D23 by folding back over the turn rather than within the turn. Since individual sharp turns at A24-N27 from various A*β* peptides are not packed too close to each other, there is an opportunity for the turns to enter the binding pocket of the Fv5E3 antibody. Nguyen et al. proposed *β*-barrel models for A*β*40 and A*β*42 in aqueous solution. The K28 residue rarely forms a salt bridge in the proposed *β*-barrel models by Nguyen et al. [57]. This model might also be potentially Fv5E3-positive.

#### The hexamer model of A*β*26-40 oligomers by Laganowsky et al

A model of the A*β*26-40 oligomer was built by Laganowsky et al. using the molecular structure of the *α*-crystalline oligomer as a template [37]. No experimental or computational work was performed to validate the direction of side chains or position of residues along the surface of model. This theoretical model has a *β*-barrel/nanotube-like structure. A nanotube is a tube-like structure with a diameter in the nanometer range. The interior diameter of the hexamer model by Laganowsky et al. is 1.1 *nm*. In this model, the residues S26 to G29 are close to its top/bottom and direct away from the interior space of the structure with a solvent-exposed K28 (Panel E of S1 Fig of SI), which creates enough space for the epitope to possibly interact with Fv5E3. However, the residues S26-G29 do not form a sharp turn in this model (a distance of 9.47 Å between the S26 and G29 C_*α*_ atoms).

The Fv model of m5E3 antibody interacts with the side, top, or bottom of the hexamer model by Laganowsky et al. in the top hundred docked complexes. In the best docked structure, Fv5E3 binds to the side of the hexamer by Laganowsky et al. with the Rosetta score of -212.33 (-2.35 per residue), which suggests a possible favorable interaction between them (Panel D of Fig 5). The formation of a salt bridge between K28 of the hexamer’s chain B, and the E102 of Fv5E3’s heavy chain may initially bring them close to each other. The complex is however not stable in the course of a 100-*ns* MD simulation (Panel F of Fig 5 in red), and the hexamer by Laganowsky et al. moves away from its initial docked position (Panel E of Fig 5).

The occupancies of hydrogen bonds formed during the simulation are low (S6 Table of SI). No ionic, aromatic-aromatic, aromatic-sulphur, or cation-*π* interactions are also present between the final conformation of Fv5E3 and the hexamer by Laganowsky et al. after 100 *ns*. The occupancies of hydrophobic interactions are also low (S7 Table of SI). The hexamer model is stable in the last 60 *ns* (Panel F of Fig 5 in blue). As the LRMSD of the complex does not converge in the course of the simulation (Panel F of Fig 5 in red), no MM-GBSA binding free energy is reported for this system.

Two other similar models of A*β* nanotubes have been proposed. Nicoll et al. provided a model of A*β* nanotubes that was reconstructed from electron microscopy images [58]. The model by Nicoll et al. did not provide enough molecular details to judge whether it could be detected by the m5E3 antibody. Yong et al. developed a computational nanotube model of A*β*40 oligomers in which the side chains of amino acid residues oriented alternately on either side of each A*β* peptide [59]. The K28 residue is solvent-exposed in this model. The curvature around the nanotube is however not sharp, so the turn at the epitope residues cannot be sharp. Individual A*β* peptides along the surface of the nanotube are also very close to each other; therefore, individual epitopes cannot enter the binding pocket of Fv5E3. Although, the coordinate file of this nanotube model was not deposited, based on its structural characteristics discussed above, it is unlikely that Fv5E3 can bind to these nanotubes specifically through its assumed epitopes.

#### The dodecamer model of A*β*42 oligomers by Gallion

A disc-shaped dodecamer model of A*β*42 oligomers was assembled by Gallion [38] (Panel F of S1 Fig of SI). As with many other computational models, the model of Gallion is not based on a single type of oligomers but rather based on different experimental data. AFM result by Ahmed et al. showed that a type of stabilized A*β*42 oligomers have a disc-shape [60]. A disc-shaped computational model based on the proposed structure by Ahmed et al. disaggregated in a 60-*ns* MD simulation [61], while the Gallion model did not [38]. Gallion’s dodecamer is composed of two hexameric disc-shaped sub-units stacked on top of each other. Each A*β* peptide was taken from the crystal structure of the tetramer model of A*β*Os by Streltsov et al. [34], with a wide solvent-accessible turn at G25-G29. The distance between G25 and G29 of individual A*β* peptides in this model is 11.51 Å. The dodecamer model by Gallion assumes that the K28 residue is buried inside, as it was shown to be buried inside the globulomers [19]. In the Gallion’s model, the K28 side chain interacts with the mainchain carbonyls of A21 and D23, and not the side chain of D23. The K28 residue is still partly solvent-exposed in some chains of the Gallion’s model.

The N-termini residues (1-16) of the model obstruct the access of Fv5E3 to the G25-G29 turn. If the N-termini residues were rigid and had little flexibility, then Fv5E3 would not be able to detect these types of oligomers specifically through its epitope. The N-termini residues of the dodecamer model were shown to be very flexible, and start to become random coil during a simulation [38]. The N-termini residues of the dodecamer are also sticking out to the solvent, and do not seem to participate in any interaction with the rest of the model as shown in Fig 1 of Ref. [38]. This suggests that their removal shall not affect the overall stability of the oligomer. In fact, the LRMSD of the dodecamer model was shown to reach a plateau status over the course of a simulation in the absence of the N-termini *α*-helices [38]. We therefore removed the N-termini residues to open up a space for Fv5E3 to bind to a turn epitope.

The top hundred docked complexes reveal that Fv5E3 binds around the “discs”, where the upper and lower discs meet (based on the view of the dodecamer in Panel F of S1 Fig of SI). Fv5E3 interacts in a similar way to the dodecamer in the top-ranked docked complex with the Rosetta score of -396.42 (-1.43 per residue, Panel G of Fig 5). The K28 residue is not solvent-exposed in the chain that docked to Fv5E3. The LRMSD of C_*α*_ atoms of the complex converges towards the last 50 *ns* of the simulation (Panel I of Fig 5 in red). The Fv5E3 is fairly stable during the whole simulation with minor adjustments with respect to the changes in the oligomer. (Panel I of Fig 5 in black). The oligomer is deviated 10 Å from its initial structure, and seems to be stable in the last 50 *ns* of the simulation (Panel I of Fig 5 in blue). Only the two interacting chains of the oligomer with Fv5E3 start to dissociate from the rest of the oligomer during the course of the 100-*ns* simulation (Panel H of Fig 5). Multiple Fv5E3 can bind around the dodecamer, and may destabilize the other parts of the oligomer. A follow-up study with a simulation with multiple Fv5E3s can confirm this prediction.

A broad range of residues in the chain D of the oligomer (residues 18 to 33) form hydrogen bonds with Fv5E3, as the chain D unfolds upon interaction with Fv5E3. The residues of chain U form hydrogen bonds only with the framework residues of Fv5E3. The assumed epitope residues in chain U participate in low occupancy hydrogen bonds. The hydrogen bonds with the highest occupancies are between E22 of chain D and D23 of chain U of the oligomer, and R96 and R46 of the light chain of Fv5E3, respectively (S6 Table of SI). There are also many low occupancy hydrophobic and aromatic-aromatic interactions between the oligomer and Fv5E3 (S7 Table of SI). These interactions further stabilize the interaction between the oligomer and Fv5E3. There is no aromatic-sulphur or cation-*π* interaction between the oligomer and Fv5E3. The average MM-GBSA binding free energy of the oligomer and Fv5E3 is -25.18 kcal/mol (std. dev. of 5.61), which also suggests the existence of a favorable interaction. The most favorable interactions contributing to the binding free energy are between R96 and R46 of the light chain of Fv5E3, and E22 of chain D (-16.49 kcal/mol) and D23 of chain U (-11.3 kcal/mol) of the oligomer, respectively.

### Interaction between Fv5E3 and models of A*β* fibrils

Various structural models have been proposed for A*β* fibrils [28, 40, 62–64]. A common structural signature of the A*β* fibrils is the cross-*β* structural motif. It is characterized by *β*-sheets running parallel along a fibril axis with *β*-strands oriented perpendicular to the axis. Each fibril may be formed from two or more cross-*β* sub-units [39, 64]. The *β*-sheets of the cross-*β* sub-units are formed of repetitive individual A*β* peptides, which create a general crowding around the m5E3 epitope. This crowding around the m5E3 epitope by itself may impede the binding of m5E3 to fibrils. The m5E3-specific epitope can also be sequestered among cross-*β* sub-units. This provides a second barrier for m5E3 binding to fibrils specifically through its epitope. To verify these claims computationally, we perform detailed analyses of the interactions between available structural models of the A*β* fibrils and the Fv model of the m5E3 antibody.

#### The model of A*β*40 fibrils by Lu et al

Lu et al. resolved with nuclear magnetic resonance (NMR) experiments a quaternary molecular structure for the A*β*40 fibrils seeded with the brain-derived A*β* fibrils [27] (pdb entry 2m4j, Panel A of S2 Fig of SI). The deposited structure of the protofilament has three layers (4.8 Å apart from each other) perpendicular to the axis of the fibril. Each layer has a three-fold symmetry of the A*β*40 peptide.

In our analyses, to better represent a fibrillar surface, we aligned three of the protofilaments along the fibrillar axis to make a longer structure with nine layers. This longer model has now meaningful ends, and a fibrillar surface between the two ends. The use of a much longer model of the fibril may not be feasible in silico, and does not seem to provide us with more insights compared to the model with nine layers. It is also not possible to perform our simulations with an infinite layer fibril using periodic images [65], as Fv5E3 can be docked to the end of the fibril. Unlike many other models of A*β* fibrils, the N-terminal residues of A*β* in this model were shown to be a part of the quaternary structure. In this structure, the distance between G25 and G29 is 10-12 Å, and thus the residues G25-G29 do not form a sharp turn. The wide turn by the epitope residues removes the conformational restriction on the side chain of K28 to reorient towards D23 for the formation of a salt bridge from within the turn, and thus not to be solvent exposed. In this model of the A*β* fibril, many adjacent epitopes are aligned closely along the axis of the fibril, and thus preventing Fv5E3 from accessing an individual epitope. The N-termini residues additionally block Fv5E3’s access to the epitope residues.

**Fig 6:**
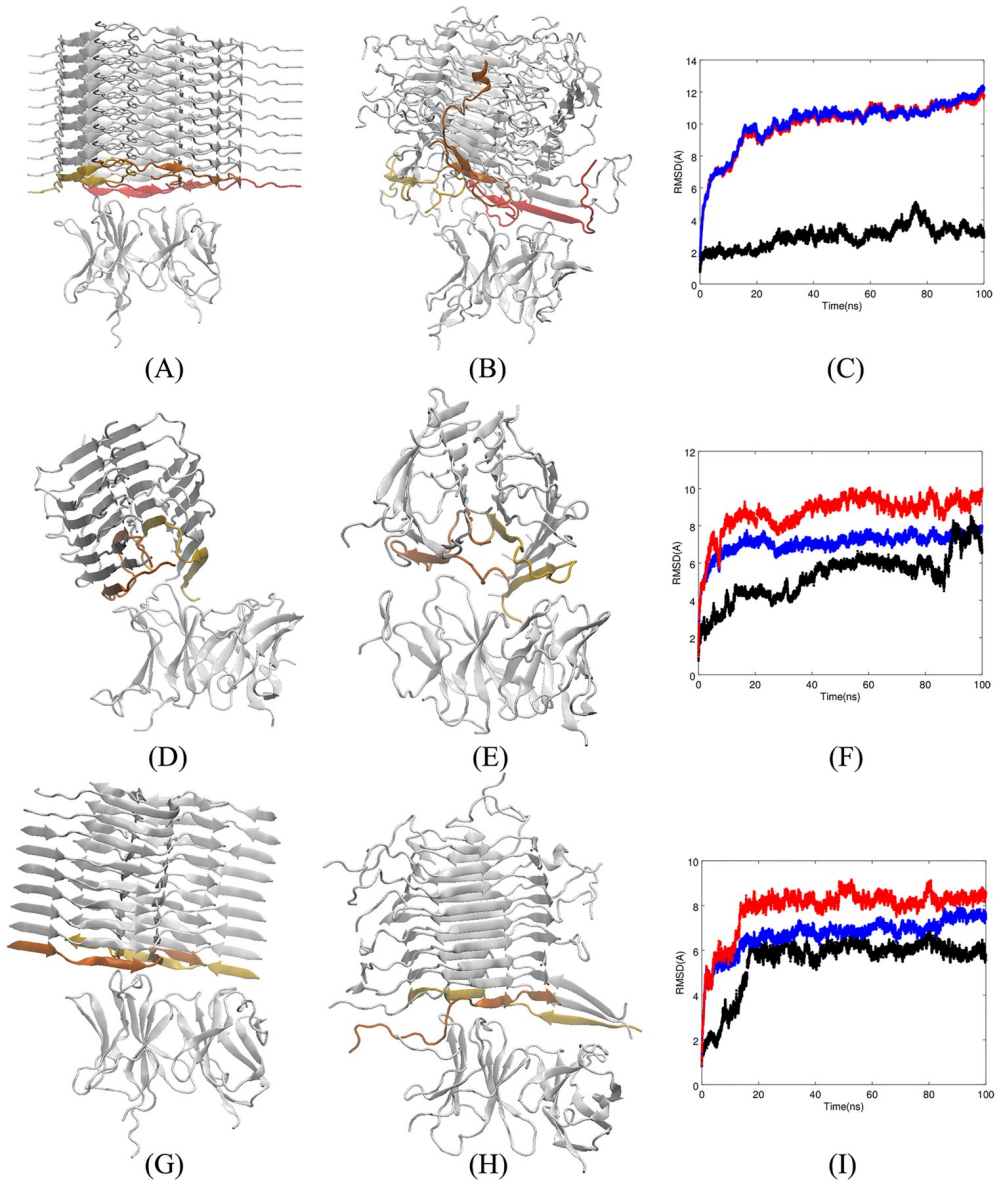
**A**) Top-ranked docked structure of Fv5E3 and the model of A*β*40 fibrils by Lu et al. The leading chains of the fibril are shown in yellow, orange and red. **B**) Complex after 100 *ns* of MD simulation. **C**) The LRMSD of C_*α*_ atoms of the complex (red), Fv5E3 (black), and the fibril (blue) during the 100 *ns* MD simulation. **D**) Top-ranked docked structure of Fv5E3 and the two-fold symmetry model of A*β*40 fibrils. The leading chains of the fibril are shown in yellow and orange. **E**) Complex after 100 *ns* of MD simulation. **F**) The LRMSD of C_*α*_ atoms of the complex (red), Fv5E3 (black), the fibril (blue) during the 100-*ns* MD simulation. **G**) Top-ranked docked structure of Fv5E3 and the zipper-like model of A*β*42 fibrils. The leading chains of the fibril are shown in yellow and orange. **H**) Complex after 100 *ns* of MD simulation. **I**) The LRMSD of C_*α*_ atoms of the complex (red), Fv5E3 (black), and the fibril (blue) during the 100-*ns* MD simulation.

To validate our prediction for the low affinity of Fv5E3 to this model of the A*β* fibril, we performed docking of this fibrillar model and Fv5E3. In the top-ranked docked structure with the Rosetta score of -828.69 (-0.76 per residue), Fv5E3 is bound to the end of the fibril and not to the surface between the two ends (Panel A of Fig 6). In the top hundred docked complexes, Fv5E3 shows a similar mode of binding only to the end of the fibril.

We further examined the stability of the top-ranked complex in a 100-*ns* MD simulation. The LRMSD of the complex does not converge, while Fv5E3 is fairly stable (Panel C of Fig 6 in red and black, respectively), and stays bound to the end of the fibril in the course of the simulation (Panel B of Fig 6). The model of the fibril however shows significant deviation of its N-terminal residues from their initial conformations, and starts to lose the A*β* peptides from its two ends (Panel B of Fig 6, and Panel C of Fig 6 in blue). The loss of the peptides at the ends of the fibril is not due to the presence of the antibody, as it happens at both ends and has been shown to happen in simulation of the fibril by itself especially if the number of layers in the model of the fibril is small [65].

Among many hydrogen bonds observed during the simulation, none are involved the epitope residues G25-G29 (S8 Table of SI). There are also many hydrophobic interactions, salt bridges and aromatic-aromatic interactions between Fv5E3 and the model of the fibril by Lu et al. Again, no epitope residues are present in any of these interactions (S9 Table of SI). R50 and K59 of Fv5E3’s heavy chain form salt bridges simultaneously with both E22 and D23 of chain V of the fibril (S8 and S9 Tables of SI). R50 and K59 are the immediate residues before and after the CDR2 of m5E3. The residues close to CDRs may participate in recognition of a cognate. R66 of the light chain of Fv5E3 forms a salt bridge with E11 of chain V of the fibrillar model. R50, K59 and R66 do not form salt bridges with A*β*Os, while participate in salt bridge formation with the fibril. Mutating them to acidic residues or glycine residues possibly can prevent their interaction with the model of the fibril by Lu et al.

As the LRMSD of the complex does not converge, it is not possible to properly measure the MM-GBSA binding affinity between Fv5E3 and this fibrillar model. As Fv5E3 interacts only with the end of the fibril, and considering the very long size of the fibrils, built of many A*β* peptides, m5E3 should have a low affinity to this type of fibrils in vitro.

A similar model with three-fold symmetry was also provided by Paravastu et al. for synthetic A*β*40 fibrils (pdb entries 2lmp and 2lmq) [64]. These fibrils were generated under the quiescent growth protocol with intermittent sonication during growth. The NMR data suggested a disordered N-terminal segment up to residue Y10. The residues 11-22 and 30-39 formed *β*-strands, and residues D23-G29 formed a turn between the two *β*-strands. The dipole-dipole couplings between K28 N*ζ* and D23 C*γ* of these fibrils were shown to indicate a 5 Å distance between them [64]. A solvent-separated salt bridge interaction between K28 and D23 were proposed to occur [64]. The twenty deposited models (pdb entries 2lmp and 2lmq) rarely show the presence of salt bridges between D23 and K28. It is possible that the intermittent sonications during the growth of the fibrils break these salt bridges. K28 is anyway buried between the *β*-sheets in these models. As the structure of the fibrillar model by Paravastu et al. is very similar to the one resolved by Lu et al. discussed above, Fv5E3 may again only bind to the end of the fibrils by Paravastu et al.

#### The model of A*β*40 fibrils by Petkova et al

A quaternary structure of synthetic A*β*40 fibrils was resolved by Petkova et al. using NMR data [28] (pdb entries 2lmn and 2lmo, Panel B of S2 Fig of SI). The preparation of these A*β* fibrils was performed with gentle agitation. Petkova et al. proposed a two-fold symmetric model for the A*β*40 fibrils. The first eight structurally disordered residues of each A*β* peptide are not present in the model. The side chains of K28s almost always form salt bridges with D23s of ±2 neighboring strands. From residues E22 to A30, a wide-turn is usually present. The distance between G25 and G29 of this model is 9.76-13.28 Å, which indicates the presence of a wide turn at the epitope residues. It is interesting that D23 and K28 in this model do not always form salt bridges with each other. In fact, some of the A*β* peptides have conformations at the epitope residues very similar to the conformation of cSNK (chain H in model 6 of 2lmn, and chain D in model 7 of pdb 2lmo with distances of 7.87 Å and 6.65 Å between G25 and G29, respectively). The epitope is also not always very crowded by the presence of adjacent epitopes. This may indicate a possible interaction/pathway between these fibrils and m5E3-positive A*β*Os.

The interaction between the fibril and Fv5E3 in the top-ranked docked complex occurs at the end of the fibril with the Rosetta score of -375.60 (-0.97 per residue, Panel D of Fig 6). In the top hundred docked complexes, Fv5E3 is also associated with the end of the fibril. The antibody stays bound to the end of the fibril after a 100 *ns* simulation of the best-ranked docked complex (Panel E of Fig 6). The complex is fairly stable in the course of the simulation, and its LRMSD converges in the last 10 *ns* (Panel F of Fig 6 in red). The antibody goes through many minor changes to allow its continuous binding to the fibril. The small 2 Å fluctuation in LRMSD of Fv5E3 in the last 20 *ns* of the simulation occurs because of the interactions of the N-terminal residues of Fv fragment with the fibril. The model of the fibril by Petkova et al. twists around the axis of the fibril during the simulation. It however maintains its overall shape (Panel E of Fig 6, and Panel F of Fig 6 in blue). The same twisting behavior is observed when a six *β*-hairpin layer model of the fibril was simulated [65]. Due to the high stability of the fibril, Fv5E3 is not able to dissociate any of the A*β* peptides from the rest of the fibril.

Many hydrogen bond interactions are present between Fv5E3 and the fibrillar model. Some of the interactions occur with the framework residues. The framework residues N77 and D61 of the heavy chain are not part of the m5E3’s original sequence, and hence the interactions with these two residues may not be present in vitro. From the epitope residues, only S26 and N27 form hydrogen bonds (with low occupancies) (S8 Table of SI). The residue E11 of chain E of the fibril, and K59 of the heavy chain form a high occupancy ionic interaction. R66 also forms a salt bridge with E22 of the fibril (S9 Table of SI). The R50 of Fv5E3’s heavy chain and Y91 of the light chain participate in high occupancy cation-*π* interactions with Y10 and K16 of the fibril, respectively (S9 Table of SI). R50, K59 and R66 interact with the fibril, but do not interact with the A*β*Os. Site-directed mutagenesis of these residues to acidic residues or glycine residues may provide an opportunity to prevent m5E3 from interacting with this type of fibrils in vitro.

The interaction between Fv5E3 and the fibrillar model is stabilized by a combination of hydrogen bonds, hydrophobic interactions, salt bridges, aromatic-aromatic and cation-*π* interactions (S8 and S9 Tables of SI). The MM-GBSA binding free energy between the fibril and Fv5E3 is favorable (-8.59 kcal/mol with std. dev. of 6.45). No epitope residues however participate in the hydrophobic, ionic, aromatic-aromatic or cation-*π* interactions (S9 Table of SI). As the fibril is very long, consisting of many A*β* peptides, the likelihood of the m5E3 antibody to bind to only the ends of the long fibril is low. Therefore, m5E3 should have a low affinity for this type of fibrils in vitro. If this interaction however occurs, the m5E3 antibody may block further growth of the fibril.

#### The model of A*β*42 fibrils by Schmidt et al

A zipper-like model for the structure of an A*β*42 dimer in fibrils was predicted by Schmidt et al. using electron cryo-microscopy data [29] (pdb entry 5aef, Panel C of S2 Fig of SI). The distance between residues G25 and G29 in this model is 15.04 Å, which means the assumed epitope residues G25-G29 do not form a sharp turn. The K28 residues are solvent-exposed in this dimer. Schmidt et al. also provided a minimal fibrillar model with six of such dimers in which the distance between adjacent dimers is 4.7 Å. We extended that fibrillar model to one with nine adjacent dimers. This longer model is a better representative of the fibril with proper ends, and a surface between the ends.

In the top hundred docked complexes, Fv5E3 binds to the end of the fibril. The top-ranked docked complex of Fv5E3 and the fibril with the Rosetta score of -460.63 (-0.6 per residue) is shown in Panel G of Fig 6. This complex after 100 *ns* of MD simulation maintains its overall structure (Panel H of Fig 6). The N-termini residues of the fibril move closer to the *β*-sheet formed by its C-termini residues and lose their secondary structure. The leading chains of the fibril start to dissociate from both ends, which could be because the model of the fibril is formed of a limited number of peptides [65] (Panel H of Fig 6). The LRMSDs of C_*α*_ atoms of the complex, the antibody and the fibril are fairly stable during the simulation (Panels I of Fig 6 in red, black and blue, respectively). The simulation is converged in the last 80 *ns* of the simulation.

Many hydrogen bond interactions occur between Fv5E3 and the zipper-like model, but none of them are formed with the epitope residues. The hydrogen bonds with highest occupancies are between D7 of chain M, V20 of chain A, I16 of chain A of the fibril, and R96 of the light chain, S31 of the heavy chain, I28 of the heavy chain of Fv5E3, respectively (S8 Table of SI). There is one ionic interaction between D7 of chain M of the fibril, and R96 of the light chain of Fv5E3 (with high occupancy). There are a few hydrophobic interactions between Fv5E3 and the zipper-like model (not with high occupancies) (S9 Table of SI). There is also an aromatic-aromatic interaction between the F3 of chain K of the zipper-like model, and Y32 of the light chain of Fv5E3 with a low occupancy (S9 Table of SI). None of the hydrophobic, ionic, aromatic-aromatic interactions are with the assumed epitope residues (S9 Table of SI). There are no aromatic-sulphur or cation-*π* interactions between Fv5E3, and the zipper-like model after 100 *ns* of simulation.

The average MM-GBSA binding free energy between Fv5E3 and the zipper-like model of the fibril is -48.95 kcal/mol (std. dev. of 7.18). This is the most favorable binding free energy among the different A*β* aggregates considered in this work. Although, the interaction is favorable, it occurs at the end of the fibril. As the fibrils are very long, consisting of many A*β* peptides, initial binding of m5E3 to the end of the fibril is a very rare event. However, if m5E3 binds to the end of the fibril, it blocks its further growth.

### Interaction between Fv5E3 and cross-*β* sub-units of A*β* fibrils

The construction of the spine of a fibril from a single cross-*β* sub-unit may not be trivial [64, 66]. As the fibrils may grow from these minimal structures, which may be present in vivo, we included them in our analyses. We are borrowing the cross-*β* unit terminology from Ref. [64]. The cross-*β* sub-units have also been used as models for A*β*Os, and the interaction of a single-domain antibody with the end or the surface between the two ends of these cross-*β* sub-units have been examined [67].

#### The model of A*β*42 cross-*β* sub-units by Lührs et al

The protofilament of the A*β*42 fibrils resolved in an NMR experiment by Lührs et al. consists of five *β*-hairpins [39] (pdb entry 2beg, Panel D of S2 Fig of SI). The protofilament by Lührs et al. is the cross-*β* sub-unit of the fibril. The residues 1-16 in the A*β* fibril were disordered, and hence their coordinates were not resolved. The residues G25-G29 of this model are part of a wide-turn from S26 to I31 with a distance of 12.56 Å between G25 and G29. The K28s residues almost always form salt bridges with D23s of the adjacent chains, while facing the inner part of the turns. K28 can become solvent exposed in the leading chain (Panel D of S2 Fig of SI). As the epitopes are also packed close to each other, it may not be possible for Fv5E3 to detect these protofilaments by specifically binding to a small number of epitopes. Lührs et al. also proposed a model of the fibrils consisting of four strands of such cross-*β* sub-units [39]. The coordinate file of this fibrillar model is not deposited.

**Fig 7:**
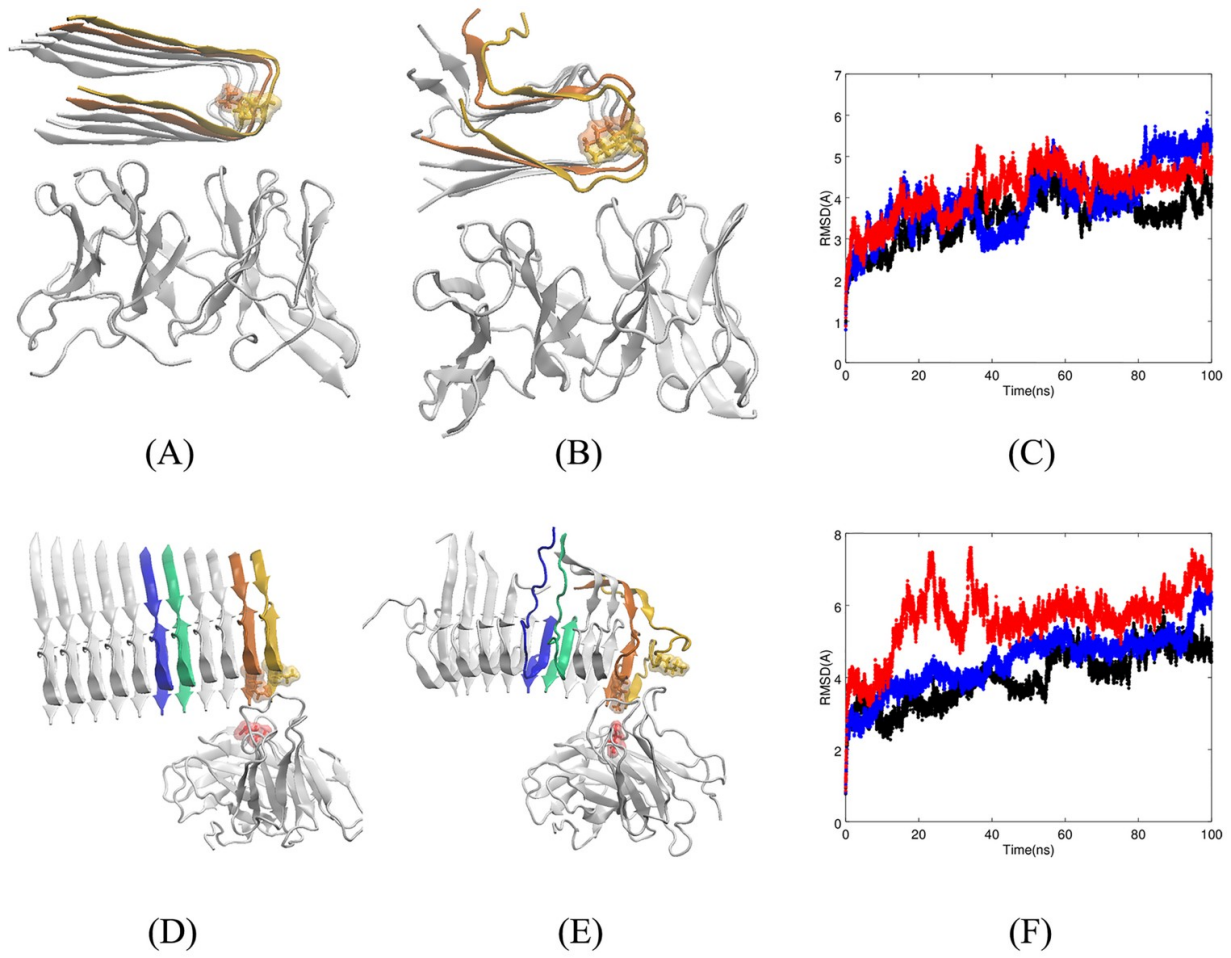
**A**) Top-ranked docked structure of Fv5E3 and Lührs’ cross-*β* sub-unit. The leading chains of the cross-*β* sub-unit are shown in yellow and orange. The K28 residue is shown in stick and solvent-exposed surface representations. **B**) Complex after 100 *ns* of MD simulation. **C**) The LRMSDs of C_*α*_ atoms of the complex (red), Fv5E3 (black), and the cross-*β* sub-unit (blue) during the 100-*ns* MD simulation. **D**) Top-ranked docked structure of Fv5E3 and Xiao’s cross-*β* sub-unit. The stick and solvent-exposed surface representations of the K28 residues of the two leading chains of the cross-*β* sub-unit, and the E102 of the heavy chain of the Fv5E3 are shown in yellow, orange and red. **E**) Complex after 100 *ns* of MD simulation. **F**) The LRMSD of C_*α*_ atoms of the complex during the 100-*ns* MD simulation.

Contrary to our prediction, in the top hundred docked complexes, Fv5E3 binds to the ends, the turn between the two *β*-sheets, or the surface of *β*-sheets between the two ends of Lührs et al. model. In the best docked complex, Fv5E3 interacts with the surface of the *β*-sheet formed by residues 17-24 with the Rosetta score of -237.08 (-2.1 per residue, Panel A of Fig 7). The antibody stays bound to the model after 100 *ns* of MD simulation (Panel B of Fig 7). This cross-*β* sub-unit goes through a lot of minor fluctuations especially between 40-80 *ns* (Panel C of Fig 7 in blue). Its LRMSD also only reaches a plateau in the last 20 *ns*. The final conformation of the cross-*β* sub-unit of the Lührs et al. (Panel B of Fig 7) after the 100-*ns* simulation is very similar to what was reported from a simulation of the model by itself [68]. The antibody is fairly stable in the presence of the protofilament. The minor fluctuations of Fv5E3 are the adjustments by the antibody to stay bound to the cross-*β* sub-unit (Panel C of Fig 7 in black). The LRMSD of the complex is converged in the last 20 *ns* of the simulation.

The high occupancy hydrogen bonds are formed with the framework residues of Fv5E3 and not its CDR residues (S10 Table of SI). The residue V24 preceding the epitope residues forms many hydrophobic interactions with the cross-*β* sub-unit (S11 Table of SI). The E22 residues of chains B, D and F form salt bridges with high occupancies (S10 and S11 Tables of SI). There are no aromatic-aromatic, aromatic-sulphur or cation-*π* interactions between the Lührs’ model and Fv5E3. The average MM-GBSA binding free energy between the Lührs’ model and Fv5E3 is favorable (-34.28 kcal/mol with std. dev. of 6.39).

Our understanding is that m5E3 may bind to such cross-*β* sub-units, if they exist in vitro or in vivo. The turn between the two *β*-sheets, and the *β*-sheets themselves are however most likely to be covered by other cross-*β* sub-units during formation of fibrils. The introduction of m5E3 in the early stages of fibrillar formation may prevent plaque formation in a susceptible individual to AD.

#### The model of A*β*42 cross-*β* sub-units by Xiao et al

The protofilament of A*β*42 fibrils resolved using NMR by Xiao et al. has an S shape [40] (pdb entry 2mxu, Panel E of S2 Fig of SI). The protofilament is the cross-*β* sub-unit of the fibril. Models of the A*β*42 fibrils have been determined with two such cross-*β* sub-units [30, 41]. In the models of the cross-*β* sub-unit and the fibril, the distance between G25 and G29 is 13.4 Å, so a sharp turn is not formed in this region. The solvent-exposed K28s form salt bridges with A42s, and not with D23s as in some other fibrillar models. The epitope residues are also located close to each other as in the other cross-*β* sub-unit or fibrillar models. Based on the above three characteristics of this model, this cross-*β* sub-unit is unlikely to be detected by Fv5E3 through its epitope residues.

In the top hundred docked complexes, Fv5E3 binds close to the epitope residues along with the C-terminal residues, or the two ends of the cross-*β* sub-unit. In the best docked complex, Fv5E3 interacts with the epitope residues and the C-terminal residues of the cross-*β* sub-unit with the Rosetta score of -496.69 (-1.41 per residue, Panel D of Fig 7). Fv5E3 after 100 *ns* of simulation stays bound to the protofilament (Panel E of Fig 7). The overall S shape structure of the cross-*β* sub-unit is maintained during the simulation (Panel E of Fig 7). Its LRMSD however does not reach a plateau (Panel F of Fig 7 in blue). The spine of the cross-*β* sub-unit bends as if it will break at some point (Panel E of Fig 7). The binding of multiple Fv5E3s to the protofilament may overcome its internal stability and lead to its fragmentation. This protofilament was shown to be stable in simulations by itself [69, 70]. A follow-up study is needed to determine if the docking of another Fv5E3 to this complex can fragment this protofilament. The perturbations seen in the LRMSD of the complex is due to the perturbations in the protofilament model, the corresponding adjustments in the antibody, and changes in their orientations with respect to each other (Panel F of Fig 7). The LRMSD of the complex does not converge during the course of the simulation (Panel F of Fig 7 in red).

An ionic interaction between K28 of chain B of Xiao’s model and E102 of the heavy chain of Fv5E3 exists with high occupancy (S11 Table of SI). Hydrogen bond and cation-*π* interaction between K28 of chain B of Xiao’s model, and Y27 of the heavy chain of Fv5E3 also exist with not high occupancy (S10 and S11 Tables of SI). The S26 epitope residue also participates in formation of hydrogen bonds with Fv5E3. However, the occupancies of its hydrogen bonds are low (S10 Table of SI). The hydrophobic interactions have very low occupancies as well. There are no aromatic-aromatic or aromatic-sulphur interactions between the cross-*β* sub-unit and the Fv model of the antibody after the 100-*ns* simulation. As the simulation does not converge (Panel F of Fig 7 in red), it is not possible to measure the binding affinity of the Fv5E3 and the cross-*β* sub-unit properly. It would be interesting to apply our approach to the model of antibodies specific to these protofilaments to see if they have the same effect of fragmenting them.

As the occupancy of most interactions between Fv5E3 and Xiao’s cross-*β* sub-units are low, we do not expect Fv5E3 to bind the fibrils consisting of such cross-*β* sub-units [30, 41] (pdb entry 2nao, Panel F of S2 Fig of SI) with high affinities.

## Discussion

In this paper, we used a combined docking and molecular dynamics approach to describe the possible interactions between the A*β*Os and the m5E3 antibody at the molecular level. Our goal was also to show why the m5E3 antibody has a low affinity for the A*β* fibrils. We proposed a molecular structural Fv model for the m5E3 antibody, which explains to a large extent its expected behavior. By using this model, we classified the A*β* aggregates as Fv5E3-positives and possibly Fv5E3-negatives.

First, we explained how Fv5E3 detects its target cyclic mimotope. We showed that the basic lysine residue of cSNK is attracted to the acidic residue E102 in CDR3 of the heavy chain of Fv5E3 (Fig 3). The initial detections of the A*β* aggregates by Fv5E3 also usually occur through the electrostatic interactions (Panels A and D of Fig 4, Panels A and D of Fig 5, and Panel D of Fig 7). However, we demonstrated that the stability of the interactions between Fv5E3 and its cognates are maintained by the high occupancy hydrogen bonds, ionic, cation-*π*, and hydrophobic interactions that all the epitope residues form with Fv5E3. Specifically, K5 and G6 of cSNK act as anchors, and form high occupancy ionic interactions and hydrogen bonds with the residues of Fv5E3 (S2 and S3 Tables of SI). In the case of some A*β* aggregates, the residues N27 and K28 act as anchors in their interactions with Fv5E3 (S4, S5, S6 and S7 Tables of SI).

The acidic residues of Fv5E3 were among the residues which provided the most favorable contributions to the pairwise decomposition of the binding free energy for all A*β*Os with the exception of the dodecamer by Gallion. Note that in the latter case the oligomer was unfolded. This indicates that Fv5E3-positive oligomers may represent basic residues on their surface, unlike some fibrils that were shown to have an anionic surface [53]. In an in silico study, the most important epitope residues for recognition of A*β* by non-oligomer specific antibodies were shown to be Phe, Glu and Asp [71]. Some of the oligomeric models discussed in this paper have cationic surfaces. Specifically, the tetramer model of the A*β*Os by Streltsov et al. has two cationic surfaces (this model is classified as Fv5E3-positive). The octadecamer by Gu et al. has anionic surfaces, a hydrophobic surface, and surfaces with a mixture of these and basic residues (the model is most likely Fv5E3-negative). The trimer by Kreutzer et al. has two hydrophobic surfaces and three cationic corners, and it is Fv5E3-positive. The top and bottom surfaces of the hexamer by Shafrir et al. are made of basic and hydrophobic residues, and the model is Fv5E3-positive. The hexamer by Laganowsky et al. is made of mainly hydrophobic surfaces (the model is most likely Fv5E3-negative). The dodecamer by Gallion has a cationic surface around the oligomer, and two anionic surfaces on top and bottom (it is classified as Fv5E3-positive).

Some Fv5E3-positive A*β*Os are recognized by Fv5E3 similar to the mimotope (Panels A-B and D-E of Fig 4, and Panels A-B of Fig 5). The affinity of Fv5E3 for different Fv5E3-positive A*β*Os is in the same range (S1 Table of SI). These A*β*Os have sharp turns at the epitope residues. There is also enough distance between adjacent epitopes of A*β*Os. Interestingly, all models of the various A*β*Os studied in this paper have some solvent-exposed K28 residue. (S1 Table of SI). The inspection of the top hundred docked complexes of Fv5E3 with the dodecamer by Gallion, and the cross-*β* sub-unit by Lührs et al. suggests that the backbone of the GSNKG turn by itself can be an epitope for Fv5E3, as the K28 residues are not solvent-exposed in the chain interacting with Fv5E3. The unexpected hydrophobic interactions of Fv5E3 and the A*β*Os are reminiscent of the KW1 antibody fragment [72] or ScFv AS [73] mechanism for the detection of A*β*Os (S5 and S7 Tables of SI).

The binding stoichiometry of the A*β*Os and the Fv model of the m5E3 antibody depends on the particular model of A*β*Os. For example, in the case of the hexamer by Shafrir et al. [36], the stoichiometry is a one-to-one stoichiometry (Panel A of Fig 5). For the tetramer model of A*β*Os by Streltsov et al. [34], two Fv5E3 can bind to a single oligomer. Another Fv5E3 can bind to the superior side of the tetramer shown in Panel D of Fig 4. For the trimer by Kreutzer et al. [33], three Fv5E3 can bind to a single oligomer. Each Fv5E3 can bind to a corner of the triangle-shaped oligomer as seen from Panel A of Fig 4. A higher stoichiometry may allow the use of lower concentration of m5E3 in vivo for neutralizing such oligomers.

The A*β* fibrils described in Refs. [27, 28, 30, 41], and [64] are unlikely to be detected by the m5E3 antibody through a small number of epitopes as many epitopes are packed very close to each other (S1 Table of SI), and are completely buried among the cross-*β* sub-units. In all models of the A*β* fibrils discussed here, no sharp turn is formed between the epitope residues (S1 Table of SI), and the K28 residues are often involved in salt bridges. The fact that the fibrils have a very long length, and the results of our docking simulations showing that Fv5E3 can only bind to the end of these fibrils (Panels A, D and G of Fig 6) explain why m5E3 has a low affinity for fibrils. As Fv5E3 binds specifically to the end of fibrils, not to their extended solvent exposed hydrophobic surfaces, its binding cannot be considered as an entropy-driven process at physiological temperatures [74]. The Fv5E3 antibody may not be able to bind to the synthetic A*β*42 fibrils described in Refs. [41] and [30] with high affinity as the occupancies of the interactions between their corresponding cross-*β* sub-units proposed by Xiao et al. [40] and Fv5E3 are low. Based on our results, we believe that the Lührs’ model of the A*β*42 cross-*β* sub-unit [39] may bind to the m5E3 antibody in vitro (Panels A-C of Fig 7). If the cross-*β* sub-unit by Lührs et al. exists in vivo, introduction of m5E3 to an individual susceptible to AD early enough can prevent the fibrillar formation and consequently deposition of plaques.

The residues R50 and K59 of the heavy chain and R66 of the light chain of Fv5E3 interact with the fibrillar models proposed by Lu et al. and Petkova et al. (S8 and S9 Tables of SI). In addition, the residues R50 and K59 interact with the cross-*β* sub-unit of Lührs et al. (S10 and S11 Tables of SI). None of these residues interact with the A*β*Os. Mutating these residues to acidic residues or glycines may prevent the possible interaction of m5E3 with these A*β* aggregates.

Our focus in this work was to determine how m5E3 detects its A*β*O cognates, and why it has low affinities for A*β* fibrils. Our computational model for the Fv portion of the m5E3 antibody explains to a good extent the molecular principle of the interaction of this oligomer-specific antibody with the cognate A*β*Os. It also explains why m5E3 cannot have the same affinity for the A*β* fibrils. It however came to our attention that for some models of A*β* aggregates, Fv5E3 seems to have a disaggregation property to dissociate individual A*β* peptides (Panels E and H of Fig 4, and Panels B and H of Fig 5), or a fragmentation property to break the spine of the cross-*β* sub-unit (Panel E of Fig 7). The same fragmentation could be seen for a longer model of the cross-*β* sub-unit by Lührs et al. with more A*β* peptides. This finding is a proof of principle for the final effectiveness of the immunotherapeutics approaches with the oligomer-specific antibody m5E3. To confirm this finding in silico, a follow-up study with longer simulations and simulations of individual A*β* aggregates shall be taken. Column chromatography or fluorescence correlation spectroscopy [75] can also validate whether the cognate A*β* aggregates disaggregate or fragment in the presence of the m5E3 antibody in vitro. Our work also demonstrated the presence of an A*β*O-specific epitope, which should be targeted by m5E3 before the disease develops to its late stages [32].

The approach developed in this paper for m5E3 can also be used for other A*β*O-specific antibody fragments, for example KW1 [72], or monoclonal oligomer-specific antibodies, for instance 204 [76], to elucidates how those antibodies detect their cognates. Recently, A*β*Os with *α*-sheet content has been characterized [77]. When a structure for these A*β*Os becomes available, it would also be interesting to assess whether they are Fv5E3-positive.

## Methods

### Antibody homology modeling

Antibodies have a Y-shaped structure. Each arm of an antibody is made of two chains of amino acids, and can bind an antigen individually. The two chains depending on their lengths are called heavy and light. The binding region of an antibody (arms of Y) is called Fab. Each Fab itself is made of a constant and an Fv fragment. Fv is the immunogenic region of Fab [78]. The regions that show most dissimilarity within Fvs are the loop regions, also called CDRs [79]. Each Fv has three CDRs. Within each class of antibodies less variable residues form a framework for that immunoglobulin superfamily.

A homology Fv model of the m5E3 antibody was built with the *Antibody* module of Rosetta software [46]. The module predicts an Fv model by homology. The conformation of the CDR3 of the heavy chain is constructed *de novo* as it is usually difficult to determine its structure solely based on its sequence. The orientations of the two chains of the framework at their interface are also adjusted accordingly. The brief descriptions of explicit Rosetta parameters used to generate this homology model are as follows (the parameters are given in italic type in parenthesis). The number of decoys to be generated (*nstruct*) was set to 2,000. A BLAST search was performed by Rosetta to find homologous templates for the framework of the antibody and each of the CDRs except CDR3 of the heavy chain. The CDRs for the light chain of the antibody and CDRs 1 and 2 of the heavy chain were grafted onto the model (*antibody_modeler*). The cysteine residues were assumed to form disulfide bonds (*find_disulf* and *norepack_disulf*). Minimization was performed on all CDR loops except the CDR3 of heavy chain (*relax_cdrs* and *freeze_h3*). A conformation for the CDR3 of the heavy chain was predicted (*h3, build_loop*, *loop_frags*, *max_frags*, and *H3_filter*). The cyclic coordinate descent method was used to adjust the dihedral angles from the N-terminus of the CDR3 of heavy chain to its C-terminus to make sure the C-terminus residue of the CDR attaches to the framework (*ccd_closure*) [80]. The extra rotamers for aromatic residues’ *χ*1 and *χ*2 dihedral angles were used during the repacking of the side chains orientations (*ex1aro, and ex2aro_only*). The side chains of the framework of the antibody were not perturbed (*norepack_antibody* and *unboundrot*). Backbone minimization was performed on CDR3 of the heavy chain along with the two flanking residues on either side of the CDR3 of heavy chain (flank_relax 2). A final round of minimization was performed on the whole structure.

The above parameters are combined in the following command for the Rosetta software: *rosetta.gcc aa M5E3 _ -s M5E3 -nstruct 2,000 -antibody_modeler -quiet -h3 -H3_filter -ex1aro -ex2aro_only -find_disulf -norepack_disulf -norepack_antibody -unboundrot -use_pdb_numbering -ccd_closure -loop_frags -build_loop -compute_hbond -max_frags 350 -relax_cdrs -freeze_h3 -flank_relax 2*.

### Antibody docking

Docking of the antibody and its A*β* cognates were performed with the Rosetta *AntibodyDock* module [47]. This flexible docking algorithm optimizes the rigid-body position of the antibody-antigen interface, the orientation of the light and heavy chains of the antibody, and the conformations of the six CDRs during each docking. Some of the details of the algorithm are summarized with the following description of the docking parameters. The number of generated decoys was 2,000 (*nstruct*). One of the two docking partners was set to be an antibody (*fab1*). The second partner was an A*β*O, a cross-*β* sub-unit or a fibril. The starting structure was initially perturbed 3 Å along the center line, 8 Å along the plane perpendicular to the center line, and rotated 8° around the center lines (*dock_pert*). The two docking partners were rotated around their center lines (*spin*). Random moves were performed a maximum of fifty times to find a Monte Carlo acceptable decoy (complex of the two partners), along with a minimization after each set of random moves to optimize the orientation of the repacked side chains at the interaction interfaces (*dock_mcm*, and *dock_rtmin*). The side chain rotamers from the initial structure were included in the prepacking step (*unboundrot*). The orientations of the light and heavy chains were adjusted with respect to each other during each of the fifty trials (*snugdock*). The CDR2 and CDR3 of the heavy chain were also perturbed and minimized during the fifty attempts to find an acceptable decoy (*snugloop, snugh3*, and *snugh2*). The predictions at these two CDRs were assumed to have the largest of deviations from their native conformations. Disulfide bonds were formed between the cysteine residues with no additional repacking (*find_disulf* and *norepack_disulf*). Multiple computer processes were used to perform the dockings (*multiple_processes_writing_to_one_directory*).

The Rosetta command used in docking simulations was *mpiexec -n 128 rosetta.gcc aa M5E3 _ -dock -dock_mcm -quiet -nstruct 2000 -fake_native -fab1 -pose -ensemble1 1 -dock_pert 3 8 8 -spin -ex1 -ex2aro_only -unboundrot -s M5E3 -dock_rtmin -find_disulf -norepack_disulf -use_pdb_numbering -fake_native -skip_missing_residues -pose -snugdock -snugloop -snugh3 -snugh2 -multiple_processes_writing_to_one_directory*.

The Rosetta score approximates the free energy of the complex. It is calculated based on a combination of physical (e.g., electrostatics), empirical (e.g. hydrogen bonds) and statistical (e.g., probability of finding the torsion angles in Ramachandran space) terms [81]. A lower score indicates a more favorable docking. There was initially no direct correlation between the physical energy terms and the Rosetta score, and only recently it has been calibrated to correlate with the physical terms. The Rosetta score also is not well correlated with the stability of complexes in different proteins. The total score is the sum of Rosetta scores for each residue. We normalized the Rosetta score by the number of residues of each complex to be able to compare them with each other.

As m5E3 antibody is a conformation-specific antibody, we do not subject the models of A*β* aggregates to MD simulations before performing docking simulations. This way we make sure Fv5E3 is docked to the structures of the A*β* aggregates as proposed by their authors, and not the structure we gain based on the settings of our simulations.

### Molecular dynamics simulations

The Molecular Dynamics (MD) simulations were performed using the NAMD software [82]. Our starting Protein Data Bank (pdb) files contained individual docked complexes. Protein structure files (psf) were generated for the initial pdb files with the *psfgen* program from the NAMD package using the charmm27 force field [83, 84]. The protonation states of the side chains of ionizable residues were assigned according to pH 7. The *nter* and *cter* patches were used to make N-termini and C-termini residues. Intra-molecular disulfide bonds were generated between the interacting cysteine residues.

The VMD software [51] was used to explicitly solvate the docked complexes in a rectangular box with TIP3P water molecules. The minimum distance between any atom of the docked structure and the edge of the water box was set to 1.8 *nm* to avoid any interaction with images of the molecule. To neutralize the system, ions (Na^+^ and Cl^−^) were added using the *autoionize* plugin of the VMD package.

The parameters used for the NAMD simulations are as follows: non-bonded van der Waals interactions were smoothly turned off between 10 Å to 12 Å; the non-bonded pairlist distance was updated every 10 steps to include pairs within 14 Å; long range electrostatics were calculated at every other step using the particle mesh Ewald method, and a grid spacing of 1 Å; the SHAKE algorithm was used to constrain bonds of hydrogen atoms with a tolerance of 10e-8; each MD time step was set to 2 femtoseconds (fs).

The simulations were performed in five discrete steps:

1. Minimization:
  Energy minimization on the solvated system was achieved with 30,000 steps of the conjugate gradient minimization method. The heavy atoms were restrained during this minimization to their initial positions with a force constant of 50 kcal/(mol*Å^2^). This step was performed to remove excess energy.
2. Thermalization and NVT Equilibration:
  The temperature of the system was gradually increased by Langevin dynamics from 0 K every 5,000 steps by 50 K until 310 K was reached. The heavy atoms were restrained during this step to their initial positions with a force constant of 50 kcal/(mol*Å^2^). The length of this step was 0.2 nanoseconds (ns). During the following NPT equilibrations, the target pressure and temperature were set to 1 atm and 310 K, respectively.
3. NPT Equilibration 1:
  The heavy atoms were restrained during this equilibration to their initial positions with a force constant of 50 kcal/(mol*Å^2^). The length of the NPT equilibration was 0.2 ns. This step was performed to bring the density of the water in the simulation box close to its experimental value of 0.99367 *g*/*cm*^3^ at 310 K.
4. NPT Equilibration 2:
  The heavy atoms were restrained during this equilibration to their initial positions with a force constant of 5 kcal/(mol*Å^2^). The length of the NPT equilibration was 0.2 ns. This step was performed to gradually release the restraints on the heavy atoms.
5. NPT Simulation with no Restraints:
  For the Fv5E3-cSNK complex, which is a much smaller system compared to antibody-oligomer/fibril complexes, we performed a 30-*ns* long simulation. For all other systems, we performed 100 *ns*-long simulations to determine the mode of interaction between the antibody and A*β* aggregates.

### MM-GBSA free energy calculations

We used the MM-GBSA (Molecular Mechanics-Generalized Born Surface Area) method with default parameters to estimate the average binding free energy from each MD simulation trajectory [85]. The MM-GBSA approximates the binding free energy by using molecular mechanics equations for the conformational energy terms, generalized Born model for the polar solvation energy, and solvent-accessible surface area term for the non-polar solvation energy for an A*β* aggregate, the antibody, and the whole complex. The method was developed as a part of the Amber software [86]. The MM-GBSA calculations were performed based on the individual MD trajectories for each of the Fv5E3-cognate complexes. First, the *chamber* tool of the Amber 15 package was used to convert the NAMD *psf* and *pdb* files to Amber *prmtop* and *inpcrd* formats, respectively. The command for this conversion was *chamber -top top_all27_prot_na.rtf -param par_all27_prot_na.inp -xpsf XXXX.psf -crd XXXX.pdb -cmap -p XXXX.prmtop -inpcrd XXXX.inpcrd*. The NAMD *dcd* trajectories were converted to Amber *netcdf* trajectories with the *cpptraj* tool from the Amber package. The last 10 *ns* (1,000 statistically independent frames saved at 5000-step intervals) of each trajectory was used for the MM-GBSA calculations. The trend of LRMSD was used as a measure of the convergence for each system. The standard deviation (std. dev.) of the MM-GBSA energy is also reported. As the same number of frames was used for the calculations of all the MD trajectories except the one for the Fv5E3-cSNK system, we do not report the standard error of mean. It can be obtained by dividing std. dev. by the square root of the number of statistically independent frames used in calculations. The MM-GBSA calculations were performed with the Python script MMPBSA.py [87] with the following command: *MMPBSA.py -O -i mmpbsa.in -o The_result.dat -sp ComplexHydrated.prmtop -cp ComplexDeHydrated.prmtop -rp m5e3Dehydrated.prmtop -lp CognateDehydrated.prmtop -y ComplexHydratedTrajectory.netcdf*.

### Analysis of interactions between Fv5E3 and its cognates

The hydrogen bonds formed between Fv5E3 and its targets in the course of MD simulations were monitored with the VMD software [51]. The cutoff distance of 3.5 Å was used [88]. The last frames of the trajectories were used to determine whether other possibly stable non-covalent interactions were formed between Fv5E3 and the A*β* aggregates during the course of the simulations. The protein interactions calculator (PIC) online server (http://pic.mbu.iisc.ernet.in/) [89] was used to identify these interactions. The default cutoff distance of 5 Å was used for hydrophobic interactions, 6 Å for ionic interactions, 4.5 Å to 7 Å for aromatic-aromatic interactions, 4.3 Å for aromatic-sulphur interactions, and 6 Å for cation-*π* interactions [89]. If both a hydrogen bond and an ionic interaction existed between two residues, the combined interaction was labeled as a salt bridge. After identifying the residues that were participating in these interactions, we measured the occupancy of these interactions during the simulations using the results from the *distance* module of the Wordom software [49]. The occupancy is defined as the fraction of time during each MD simulation that interactions are present in.

The LRMSD was calculated for each trajectory by aligning all the frames to the initial structure. The LRMSD was calculated using the RMSD Trajectory Tool from VMD [51].

## Supporting information

S1 Fig Structural models of A*β*Os in cartoon representation. The K28 residue is shown in both the solvent-accessible surface and stick representations. **A)** Trimer by Kreutzer et al. [33]**B)** Tetramer by Streltsov et al. [34]**C)** Octadecamer by Gu et al. [35]**D)** Hexamer by Shafrir et al. [36]**E)** Hexamer by Laganowsky et al. [37]**F)** Dodecamer by Gallion. [38]
(TIF)

S2 Fig Structural models of the A*β* fibrils and cross-*β* sub-units in cartoon representation. The K28 residue is shown in both solvent-exposed surface and stick representations. **A)** Three-fold symmetry model of A*β* fibrils by Lu et al. [27]**B)** Two-fold symmetry model of A*β* fibrils by Petkova et al. [28]**C)** Two-fold symmetry zipper-like model of A*β* dimers in fibrils by Schmidt et al. [29]**D)** Cross-*β* sub-unit by Lührs et al. [39]**E)** Cross-*β* sub-unit by Xiao et al. [40]**F)** Model of A*β* fibrils by Wälti et al. [30]
(TIF)

S3 Fig Field lines and the projection of the electrostatic potential to the solvent-exposed surface of Fv5E3. Blue and red colors represent positive and negative values of the electrostatic potential, respectively. The settings of VMD [51] for the intensities of electrostatic fields (FieldLines) of this image are Color Scale Data Range of (-10, 10), GradientMag of 8.31, Min Length of 1, and Max Length of 200.6.
(TIF)

S4 Fig (TIF)

S5 Fig (TIF)

S6 Fig (TIF)

S7 Fig (TIF)

S1 Raw images (TIF)

S2 Raw images (TIF)

S1 Text (TXT)

S1 File (PDF)

S2 File (TEX)

S1 Table The summary of various features of A*β* aggregates. (PDF)

S2 Table The residues forming hydrogen bonds between Fv5E3 and cSNK. The word “main” stands for main chain. The word “side” stands for side chain. Occupancy is the fraction of time during the MD simulation that these interactions exist.
(PDF)

S3 Table The residues participating in ionic and cation-*π* interactions between Fv5E3 and the cSNK mimotope. (PDF)

S4 Table The residues forming hydrogen bonds between Fv5E3 and the experimental models of A*β*Os. (PDF)

S5 Table The residues participating in hydrophobic, ionic, and aromatic-aromatic interactions between Fv5E3 and the experimental models of A*β*Os. (PDF)

S6 Table The residues forming hydrogen bonds between Fv5E3, and the computational and theoretical models of A*β*Os. (PDF)

S7 Table The residues participating in hydrophobic, ionic and cation-*π* interactions between Fv5E3, and the computational and theoretical models of A*β*Os. (PDF)

S8 Table The residues forming hydrogen bonds between Fv5E3 and the models of A*β* fibrils. (PDF)

S9 Table The residues participating in hydrophobic, ionic and aromatic-aromatic interactions between Fv5E3 and the models of A*β* fibrils. (PDF)

S10 Table The residues forming hydrogen bonds between Fv5E3 and the cross-*β* sub-units of A*β* fibrils. (PDF)

S11 Table The residues participating in hydrophobic, ionic, and aromatic-aromatic interactions between Fv5E3 and the cross-*β* sub-units of A*β* fibrils. (PDF)
