# Supplementary figures and images for: Molecular interactions between monoclonal oligomer-specific antibody 5E3 and its amyloid beta cognates
Source: PLoS One. 2020 May 29;15(5):e0232266. doi: 10.1371/journal.pone.0232266 (PMC7259632; doi:10.1371/journal.pone.0232266)

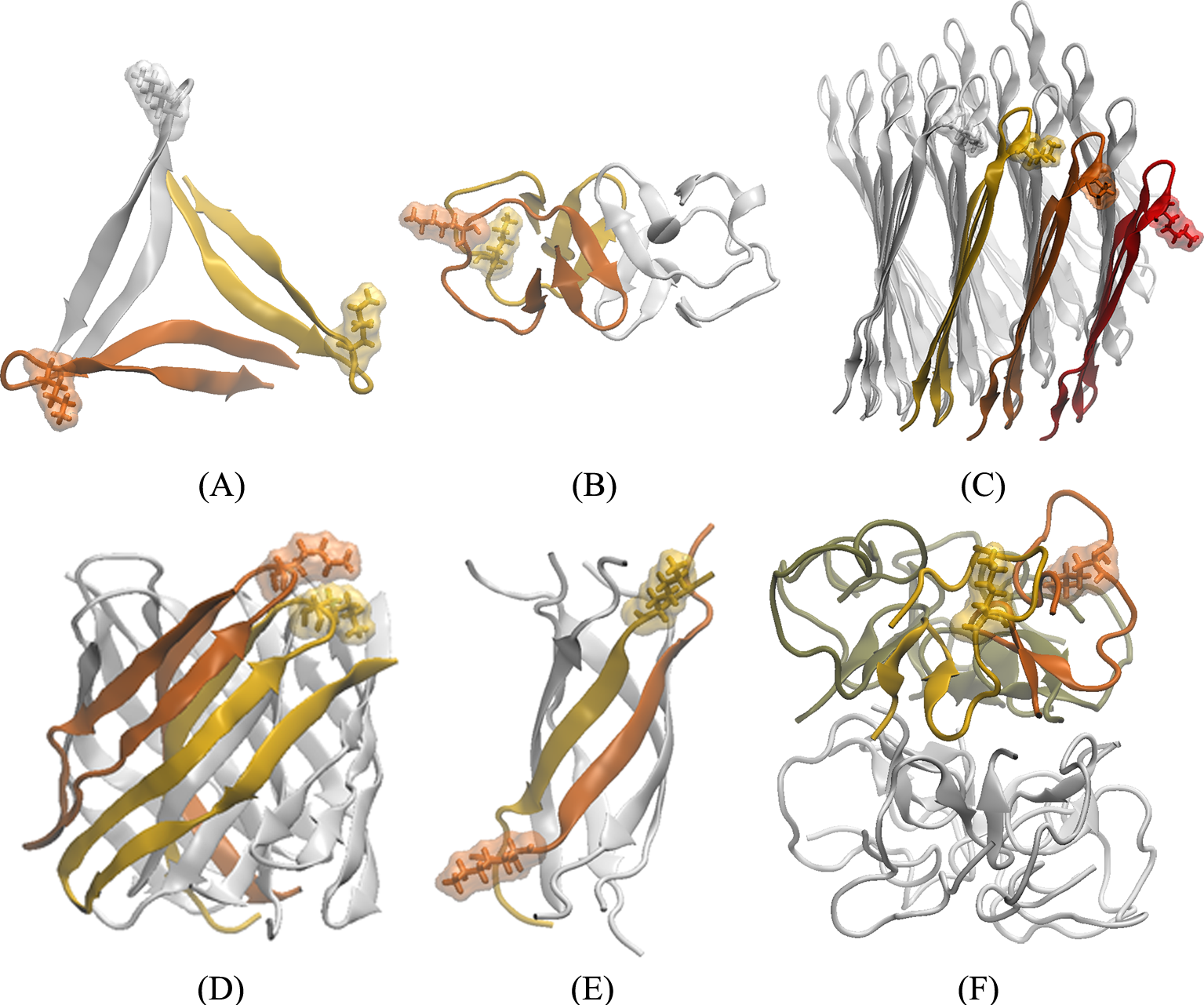

Supplement: S1 Fig — The K28 residue is shown in both the solvent-accessible surface and stick representations. A) Trimer by Kreutzer et al. [33]B) Tetramer by Streltsov et al. [34]C) Octadecamer by Gu et al. [35]D) Hexamer by Shafrir et al. [36]E) Hexamer by Laganowsky et al. [37]F) Dodecamer by Gallion. [38] (TIF) [file pone.0232266.s001.tif]

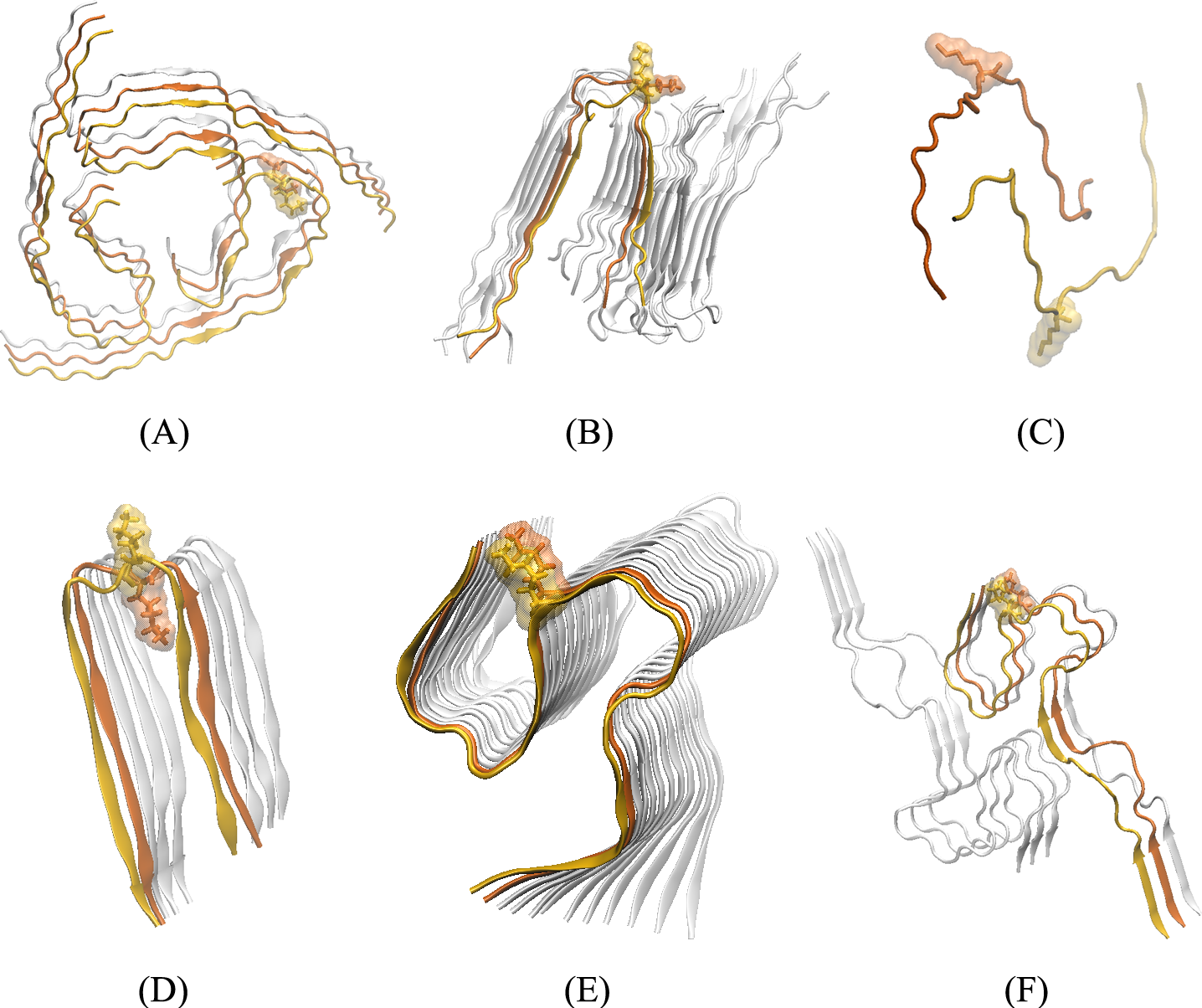

Supplement: S2 Fig — The K28 residue is shown in both solvent-exposed surface and stick representations. A) Three-fold symmetry model of Aβ fibrils by Lu et al. [27]B) Two-fold symmetry model of Aβ fibrils by Petkova et al. [28]C) Two-fold symmetry zipper-like model of Aβ dimers in fibrils by Schmidt et al. [29]D) Cross-β sub-unit by Lührs et al. [39]E) Cross-β sub-unit by Xiao et al. [40]F) Model of Aβ fibrils by Wälti et al. [30] (TIF) [file pone.0232266.s002.tif]

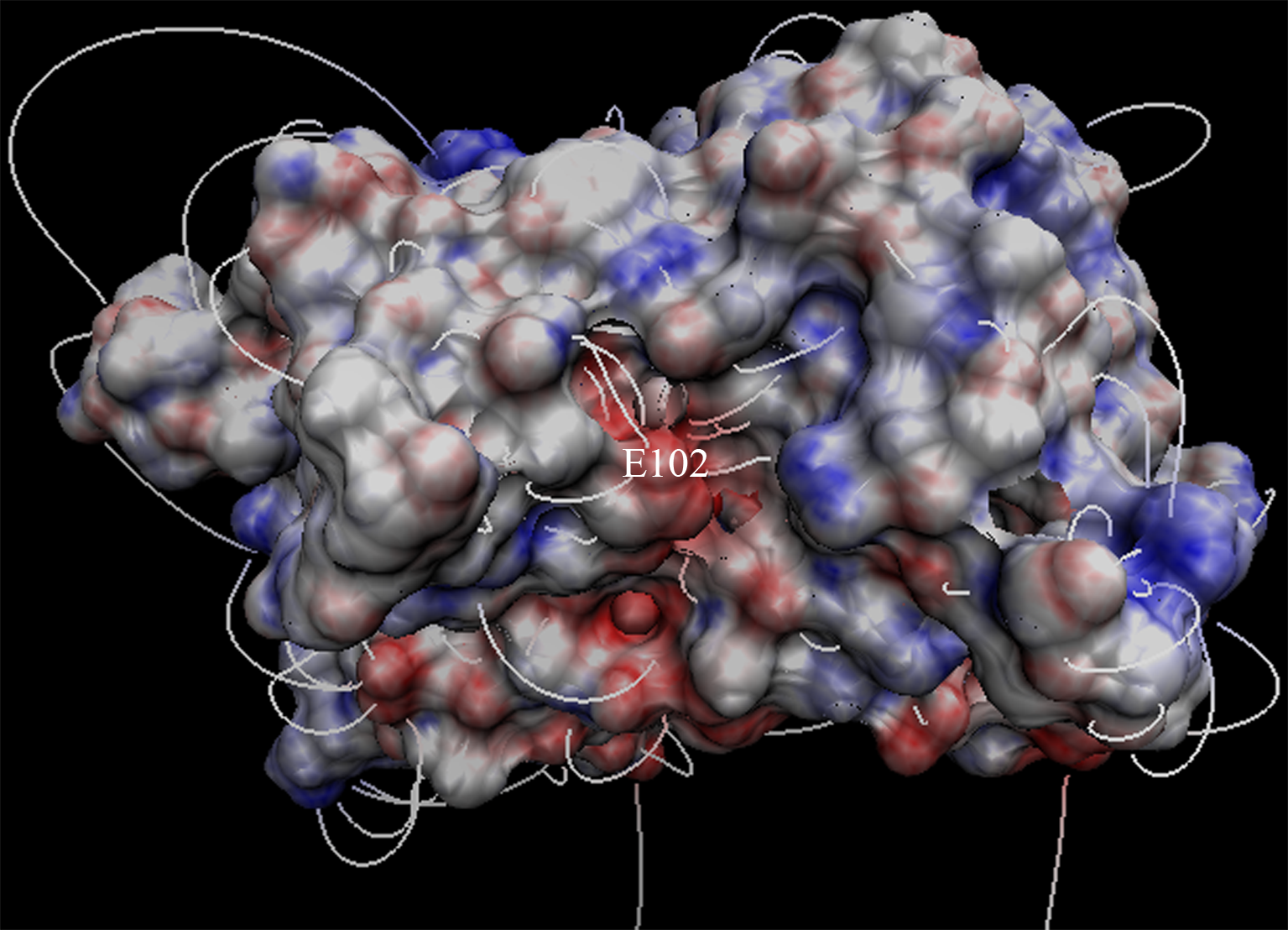

Supplement: S3 Fig — Blue and red colors represent positive and negative values of the electrostatic potential, respectively. The settings of VMD [51] for the intensities of electrostatic fields (FieldLines) of this image are Color Scale Data Range of (-10, 10), GradientMag of 8.31, Min Length of 1, and Max Length of 200.6. (TIF) [file pone.0232266.s003.tif]

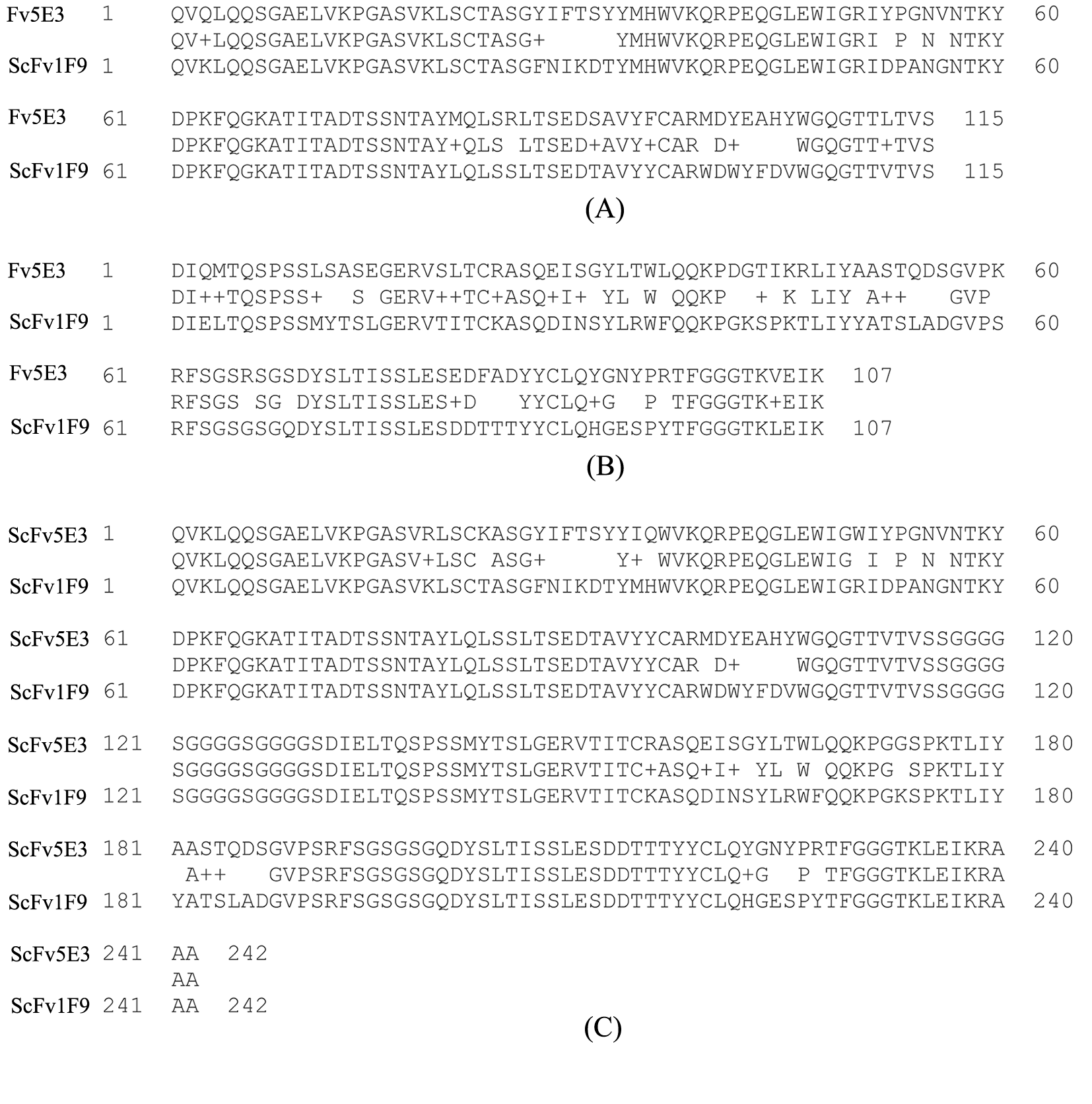

Supplement: S4 Fig — (TIF) [file pone.0232266.s004.tif]

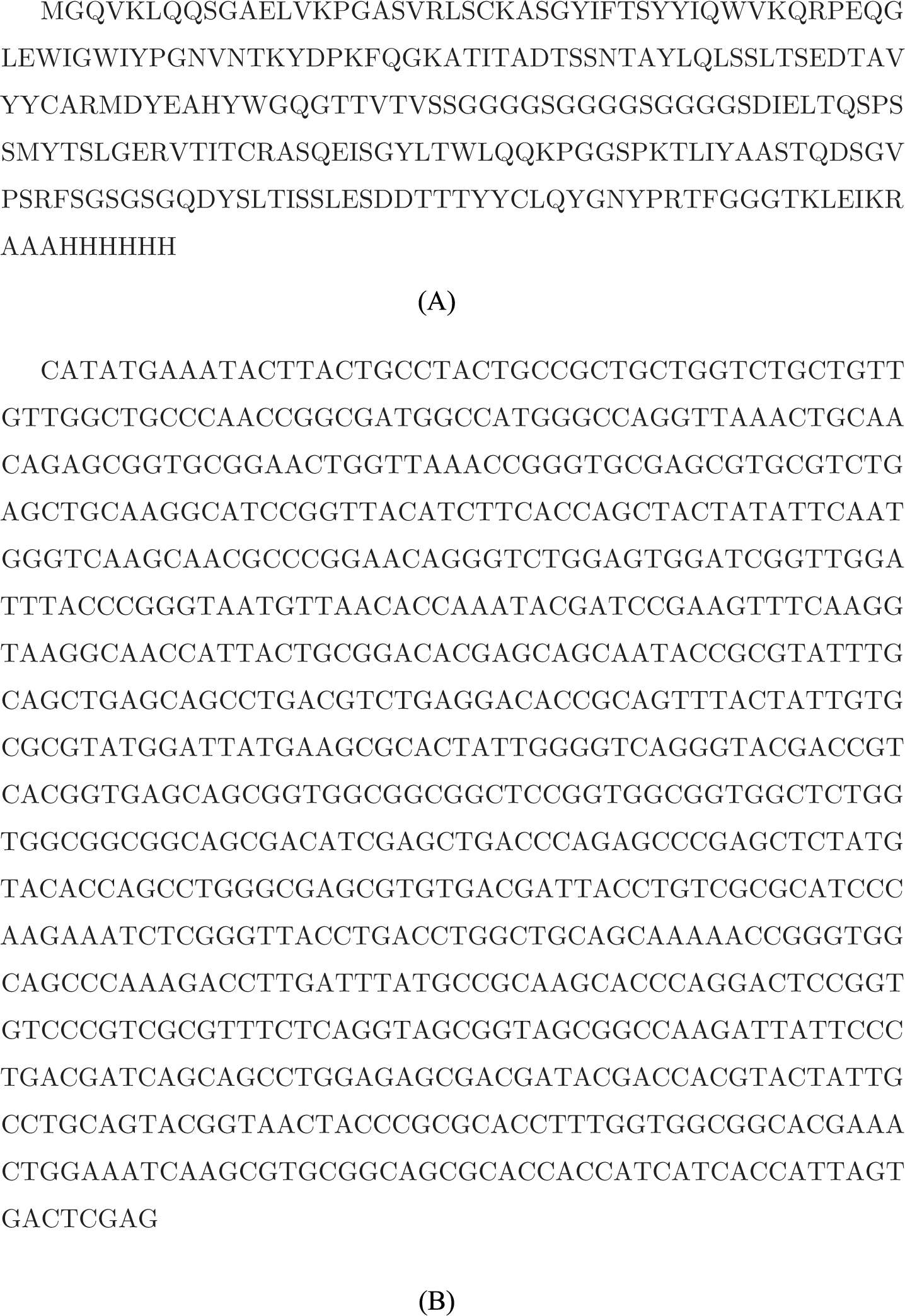

Supplement: S5 Fig — (TIF) [file pone.0232266.s005.tif]

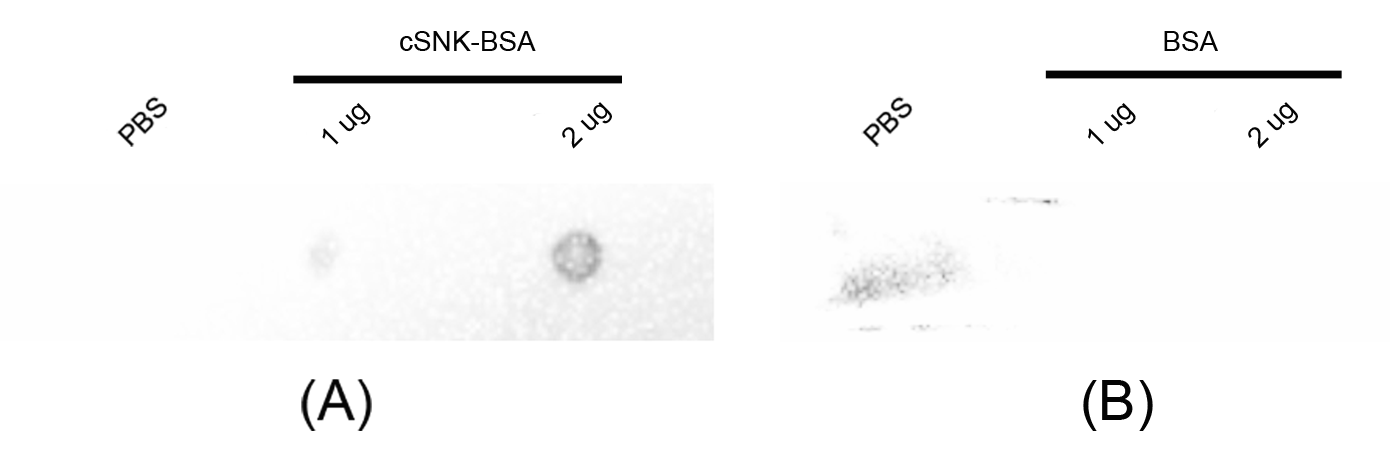

Supplement: S6 Fig — (TIF) [file pone.0232266.s006.tif]

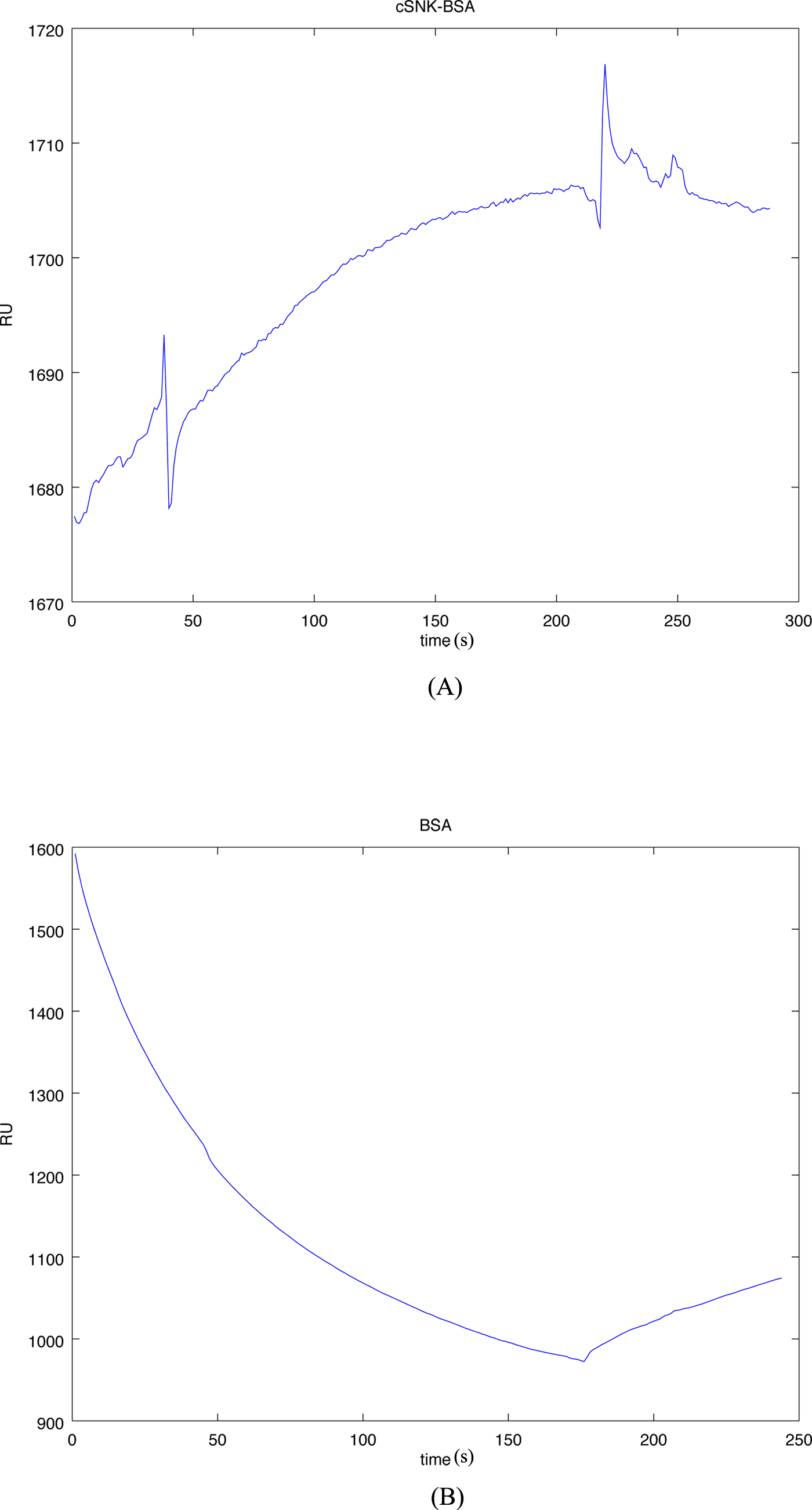

Supplement: S7 Fig — (TIF) [file pone.0232266.s007.tif]

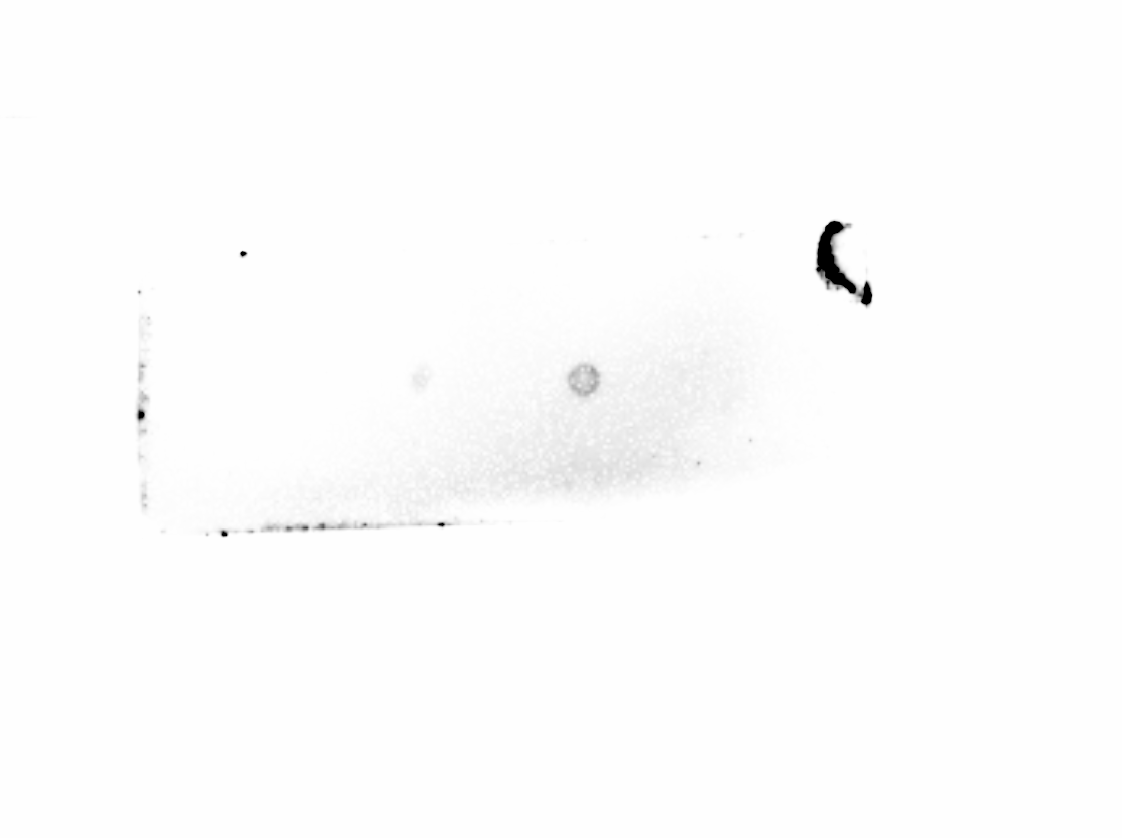

Supplement: S1 Raw images — (TIF) [file pone.0232266.s008.tif]

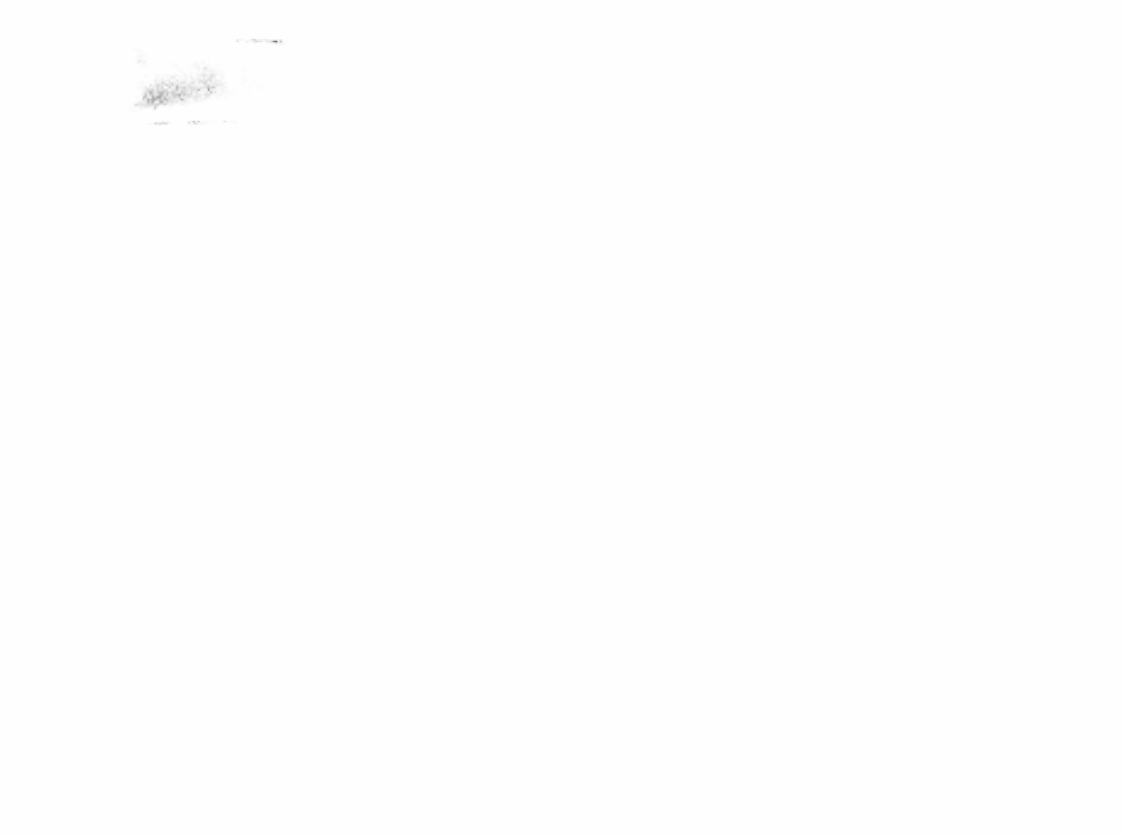

Supplement: S2 Raw images — (TIF) [file pone.0232266.s009.tif]
