# Supplementary material for: Molecular interactions between monoclonal oligomer-specific antibody 5E3 and its amyloid beta cognates
Source: PLoS One. 2020 May 29;15(5):e0232266. doi: 10.1371/journal.pone.0232266 (PMC7259632; doi:10.1371/journal.pone.0232266)
Supplement: S1 File — (PDF) [file pone.0232266.s011.pdf]

Supporting Information: Molecular Details of the Interactions between Monoclonal Oligomer-Specific Antibody 5E3 and its Amyloid Beta Cognates

Massih Khorvash[1,2◑], Nick Blinov[3,4◑], Carol Ladner-Keay[4,5], Jie Lu[2], Judith M. Silverman[2], Ebrima Gibbs[2], Yu Tian Wang[2], Andriy Kovalenko[3,4], David Wishart[4,5,6], Neil R. Cashman[1,2*¤]

1 Department of Medicine, University of British Columbia, Vancouver, British Columbia, Canada
2 University of British Columbia, Djavad Mowafaghian Centre for Brain Health, Vancouver, British Columbia, Canada
3 Department of Mechanical Engineering, Edmonton, Alberta, Canada
4 National Research Council of Canada, Edmonton, Alberta, Canada,
5 Department of Biological Sciences, University of Alberta, Edmonton, Alberta, Canada
6 Department of Computing Science, University of Alberta, Edmonton, Alberta, Canada

◑These authors contributed equally to this work.
¤Center for Brain Health, Koerner Pavilion 2211 Wesbrook Mall, Vancouver, BC Canada V6T 2B5
*** neil.cashman@vch.ca**

Experimental Verification of the CDRs of m5E3

Materials

The following reagents were used in this study: yeast extract (Fisher Scientific), tryptone (Fisher Scientific), hepes (Life Technologies), kanamycin (Fisher Scientific), isopropyl β-D-thiogalactoside (IPTG, Sigma Aldrich), urea (Fisher Scientific), bovine serum albumin (BSA, Sigma Aldrich), cSNK conjugated to BSA (cSNK-BSA, CPC Scientific), phosphate-buffered saline (Gibco®PBS, Fisher Scientific), tris-buffered saline (TBS, Fisher Scientific), Tween 20 (Fisher Scientific), anti-mouse secondary antibody (GE Healthcare), anti-His$_6$ antibody H8 (Invitrogen), SuperSignal^{TM} West Femto (Thermo Fisher). The nitrocellulose membrane was purchased from GE Healthcare.

Surface plasmon resonance (SPR) was performed on a Biacore®3000 system equipped with a Ni^{2+}-NTA (nickel-nitrilotriacetic acid) sensor chip. A degassed running buffer HBS-N [0.01 M Hepes pH 7.4, 0.15 M NaCl], and a degassed wash buffer HBS-EP [0.01 M Hepes pH 7.4, 0.15 M NaCl, 3 mM EDTA, 0.005% w/v Surfactant P20] were used to wash the biosensor chip. The standard BIAnormalizing solution (70% (w/v) glycerol) was used to normalize the system. The HBS-N, HBS-EP buffers, BIAnormalizing solution, and the Ni^{2+}-NTA chip were purchased from GE Healthcare.

Design of the Single Chain Variable Fragment of m5E3

To construct the single chain variable fragment of Fv5E3 (ScFv5E3), we used the sequence of the anti-lysozyme ScFv1F9 (pdb entry 1dzb) [1] as our template for the framework region. This ScFv had the highest sequence similarity with the heavy chain of Fv5E3. In particular, we found 82% and 66% sequence identity between the heavy and light chains of Fv5E3 and the anti-lysozyme ScFv1F9, respectively (Panels A-B

of Fig S4). We grafted the m5E3 CDRs onto the anti-lysozyme ScFv1F9 framework (Panel C of Fig S4). Rather than using a simple "cut and paste" approach we analyzed and modified the regions around the CDRs quite carefully. In particular, in situations where those residues in close proximity to the m5E3 CDRs were different from the corresponding residues of ScFv1F9, the original m5E3 residues were used instead. This was done to ensure the availability of these potentially important residues in binding of ScFv5E3 with its cognates. For the residues further away from the CDRs, the framework residues of ScFv1F9 were used to ensure the recombinant ScFv5E3 would fold properly. A 15-residue linker peptide $(GGGGS)_3$ was used to connect the C-terminus of the heavy chain to the N-terminus of the light chain. A PelB (pectate lyase B) sequence MKYLLPTAAAGLLLLAAQPAMA [2], was added to the N-terminus of the heavy chain. The PelB signal is used by proteins destined for the periplasmic space of E. coli [3]. Additionally, a His_6 tag was added to the C-terminus of the light chain to assist with purification and binding assay development. The protein sequence (Panel A of Fig S5) was then converted to a DNA sequence using known E. coli codon preferences (Panel B of Fig S5). An NcoI restriction site was engineered into the C-terminus of the PelB sequence, and NdeI and XhoI restriction sites were added to the beginning and the end of ScFv5E3, respectively. These two sites were used to insert ScFv5E3 to the pET41a expression vector. The synthetic gene for SvFv5E3 was ordered from ATUM.

The ScFv5E3 was expressed in BL21 (DE3) cells in super broth medium (3.5% tryptone, 2.0% yeast extract, 0.5% NaCl, 0.5% 1 N NaOH). The cells were induced at A_{600} of 1.5 with 1 mM IPTG at 30° C. The ScFv5E3 was extracted with the extraction buffer containing 33 mM hepes, 2 M urea, pH 8. The ScFv5E3 was purified on a Ni^{2+}-NTA column. The step dialysis was performed by exchanging buffers containing 2 M, 1 M, 0.5 M and no urea.

Figure S4. A) Alignment of the heavy chains of Fv5E3 and ScFv1F9. **B)** Alignment of the light chains of Fv5E3 and ScFv1F9. **C)** Alignment of ScFv5E3 and ScFv1F9.

Figure S5. A) Amino acid sequence of the designed ScFv5E3. Note that amino acids Met and Gly at the N-terminus are the result of introducing an NcoI site. **B)** The DNA sequence for ScFv5E3.

Examining the Binding of the Mimotope to ScFv5E3

Dot Blot Study

To verify the capability of the recombinant ScFv5E3 to bind to the cSNK mimotope of m5E3, a far dot blot was performed. The cSNK-BSA at two different amounts of 1 and 2 µg was placed on a nitrocellulose membrane along with 1 µL of PBS as a negative control. After the dots were dried, the membrane was blocked with 2% BSA in PBS with 0.1% Tween 20 (PBST) for 1 h at room temperature. The membrane was then probed with ScFv5E3 (10 µg/ mL in blocking buffer) overnight in the cold room. After three 10-min washes in TBS with 0.1% Tween 20 (TBST), the membrane was probed with the H8 antibody (1:6000 in blocking buffer) overnight in the cold room. Again, after three 10-min washes with TBST, the membrane was probed with the anti-mouse secondary antibody (1:5000 in blocking buffer) for 1 h at room temperature. The membrane was washed three times with TBST, 10 min each, and developed with SuperSignal^{TM} West Femto (Panel A of Fig S6). The same far dot blot was repeated with BSA as a negative control to make sure the ScFv5E3 was not binding to the carrier protein BSA (Panel B of Fig S6).

Figure S6. **A)** Far dot blot showing the binding of ScFv5E3 to cSNK-BSA. **B)** Far dot blot of BSA as a negative control.

SPR Study

SPR as a label-free detection method [4] was used to determine the molecular interaction of ScFv5E3 (ligand) and the cSNK-BSA (analyte). The ScFv5E3 ligand was immobilized to the surface of a Ni^{2+}-NTA sensor chip via its His_6 tag. To achieve this goal, 4 µg of the diluted ScFv5E3 sample (2 µg/ mL) was injected over the surface of the chip at the flow rate of 5 mL/min. The cSNK-BSA was detected by ScFv5E3 at the optimzed pH of 6.8 (Panel A of Fig S7), while the BSA alone did not bind to the ScFv5E3 (Panel B of Fig S7). The concentration of the cSNK-BSA and BSA were 20 µg/ mL.

Figure S7. RU stands for response unit. **A)** Binding of ScFv5E3 to its mimotope. **B)** Lack of binding of ScFv5E3 to the carrier protein BSA.

Future Work

The isolation of a specific AβO has proven to be a difficult task [5], and no NMR or crystal structure of an AβO-specific antibody with its cognates have been published yet [6]. To the best of our knowledge, no calorimetry or SPR studies have been performed on the experimental models discussed in this paper [7–9] with any AβO-specific antibody. As some of the Fv5E3-positive AβOs in this paper have already been purified to resolve a structure for them, they might prove to be good candidates for determining the structure of ScFv5E3 in the bound state.

Acknowledgments

The use of the Biacore®3000 facility at the Michael Smith Laboratory at University of British Columbia, Vancouver, BC is appreciated. We are gratitude to Luke McAlary and Peng Zhang for useful discussions.

References

1. Aÿ J, Keitel T, Küttner G, Wessner H, Scholz C, Hahn M, et al. Crystal structure of a phage library-derived single-chain fv fragment complexed with turkey egg-white lysozyme at 2.0 Å resolution. Journal of Molecular Biology. 2000;301(2):239 – 246.

2. Lei S, Lin H, Wang S, Callaway J, Wilcox G. Characterization of the Erwinia carotovora pelB gene and its product pectate lyase. Bacteriol. 1987;169:4379–83.

3. Yoon S, Kim S, Kim J. Secretory production of recombinant proteins in Escherichia coli. Recent Pat Biotechnol. 2010;4(1):23–9.

4. Piliarik M, Vaisocherová H, Homola J. In: Rasooly A, Herold KE, editors. Surface Plasmon Resonance Biosensing. Totowa, NJ: Humana Press; 2009. p. 65–88.

5. Doig AJ, del Castillo-Frias MP, Berthoumieu O, Tarus B, Nasica-Labouze J, Sterpone F, et al. Why Is Research on Amyloid-β Failing to Give New Drugs for Alzheimer's Disease? ACS Chemical Neuroscience. 2017;8(7):1435–1437.

6. Arai H, Glabe C, Luecke H. Crystal structure of a conformation-dependent rabbit IgG Fab specific for amyloid prefibrillar oligomers. Biochim Biophys Acta. 2012;1820(12):1908–14.

7. Kreutzer AG, Hamza IL, Spencer RK, Nowick JS. X-ray Crystallographic Structures of a Trimer, Dodecamer, and Annular Pore Formed by an Aβ17-36 β-Hairpin. American Chemical Society. 2016;138.

8. Streltsov V, Varghese J, Masters C, Nuttall S. Crystal Structure of the Amyloid-β p3 Fragment Provides a Model for Oligomer Formation in Alzheimer's Disease. Neuroscience. 2011;31(4):1419–26.

9. Gu L, Liu C, Stroud JC, Ngo S, Jiang L, Guo Z. Antiparallel Triple-strand Architecture for Prefibrillar Aβ42 Oligomers. Biological Chemistry. 2014;289(39):27300–27313.
