# Supplementary material for: Molecular interactions between monoclonal oligomer-specific antibody 5E3 and its amyloid beta cognates
Source: PLoS One. 2020 May 29;15(5):e0232266. doi: 10.1371/journal.pone.0232266 (PMC7259632; doi:10.1371/journal.pone.0232266)
Supplement: S1 Table — (PDF) [file pone.0232266.s013.pdf]

Model	Sequence	Size (number of residues in each chain of the model)	Number of chains	Rosetta score (normalized to the number of residues)	Turn's residues	Distance of G25 to G29 (Å)	Distance of adjacent K28 residues (Å)	Number of adjacent K28s	K28 direction	Stoichiometry	Binding free energy (kcal/mol)	Fv5E3-positive?
cSNK	CGSNKGC	7	1	-150.59 (-21.51)	C1-C7	5.5	-	0	solvent-exposed	1	-41.56	Yes
Kreutzer et al. model	Ornithine-Aβ17-36	21	3	-206.45 (-3.27)	G25-C29	6.6	30.15	2	solvent-exposed	3	-31	Yes
Streltsov et al. model	Aβ18-41	24	4	-247.37 (-2.51)	G25-I31	11.66	6.54	1	solvent-exposed in some chains	2	-17.10	Yes
Gu et al. model	Aβ42	42	16	-596.59 (-0.88)	G25-G29	7.8	9.87 and 12.08	6	solvent-exposed in the last layer	-	-	Most likely no
Shafrir et al. model	Aβ42	42	6	-416.88 (-1.65)	G25-G29	7.3	6.17	2	solvent-exposed	1	-30.46	Yes
Laganowsky et al. model	Aβ26-40	15	6	-212.33 (-2.35)	-	9.47	13.29	2	solvent-exposed	-	-	Most likely No
Gallion model	Aβ18-41	23	12	-396.42 (-1.43)	G25-I31	11.51	16.75 and 23.38	3	partially solvent-exposed in some chains	1	-25.18	Yes if the proposed N-termini residues are flexible
Lu et al. model	Aβ40	40	27	-828.69 (-0.76)	G25-G29	10-12	multiples of 4.8	many	burried	2	-	Maybe to the end of fibril
Petkova et al. model	Aβ40	32	12	-375.60 (-0.97)	E22-A30	9.76-13.28	multiples of 7.47	many	burried	2	-8.59	Maybe to the end of fibril
Schmidt et al. model	Aβ42	42	18	-460.63 (-0.6)	G25-G29	15.04	multiples of 4.7	many	partially solvent-exposed	2	-48.95	Maybe to the end of fibril
Lührs et al. model	Aβ42	26	5	-237.08 (-2.1)	S26-I31	12.56	4.8, 9.6	2, 2	buried except for the leading chain	1	-34.28	Yes
Xiao et al. model	Aβ42	32	11	-496.69 (-1.41)	F20-A30	13.4	4.8, 9.6	2, 2	solvent-exposed	-	-	Most likely No

Table S1. The summary of various features of Aβ aggregates.
