# Supplementary material for: Molecular interactions between monoclonal oligomer-specific antibody 5E3 and its amyloid beta cognates
Source: PLoS One. 2020 May 29;15(5):e0232266. doi: 10.1371/journal.pone.0232266 (PMC7259632; doi:10.1371/journal.pone.0232266)
Supplement: S2 Table — The word “main” stands for main chain. The word “side” stands for side chain. Occupancy is the fraction of time during the MD simulation that these interactions exist. (PDF) [file pone.0232266.s014.pdf]

cSNK residue	Fv5E3 residue	Fv5E3 chain	Fv5E3 residue position	Occupancy
K5-Side	D100-Main	heavy	CDR3	4.69%
G6-Main	Y94-Side	light	CDR3	5.20%
G6-Main	Y33-Side	heavy	CDR1	23.67%
G6-Main	R96-Side	light	CDR3	28.82%
K5-Side	M99-Main	heavy	CDR3	62.84%
K5-Side	E102-Side	heavy	CDR3	66.38%
G6-Main	G92-Main	light	CDR3	70.04%
G6-Main	D100-Side	heavy	CDR3	97.60%

Table S2. The residues forming hydrogen bonds between Fv5E3 and cSNK. The word ”main” stands for main chain. The word ”side” stands for side chain. Occupancy is the fraction of time during the MD simulation that these interactions exist.
