# Supplementary material for: Molecular interactions between monoclonal oligomer-specific antibody 5E3 and its amyloid beta cognates
Source: PLoS One. 2020 May 29;15(5):e0232266. doi: 10.1371/journal.pone.0232266 (PMC7259632; doi:10.1371/journal.pone.0232266)
Supplement: S3 Table — (PDF) [file pone.0232266.s015.pdf]

cSNK	Fv5E3 residue	Fv5E3 chain	Fv5E3 residue position	Type	Occupancy
K5	D100	heavy	CDR3	ionic	11.86%
K5	E102	heavy	CDR3	ionic	82.06%
K5	Y32	heavy	CDR1	cation-π	30.83%

Table S3. The residues participating in ionic and cation-π interactions between Fv5E3 and the cSNK mimotope.
