# Supplementary material for: Molecular interactions between monoclonal oligomer-specific antibody 5E3 and its amyloid beta cognates
Source: PLoS One. 2020 May 29;15(5):e0232266. doi: 10.1371/journal.pone.0232266 (PMC7259632; doi:10.1371/journal.pone.0232266)
Supplement: S4 Table — (PDF) [file pone.0232266.s016.pdf]

	The oligomer residue	The oligomer chain	Fv5E3 residue	Fv5E3 chain	Fv5E3 residue position	Occupancy
Trimer by Kreutzer et al.	G25-Main	B	R96-Side	light	CDR3	10.30%
	N27-Side	B	D100-Side	heavy	CDR3	11.48%
	N27-Side	B	S31-Main	heavy	CDR1	16.27%
	K28-Side	B	D100-Main	heavy	CDR3	17.86%
	E22-Side	A	S56-Side	light	framework	24.50%
	G33-Main	B	Y49-Side	light	framework	29.25%
	D23-Side	B	Y49-Side	light	framework	33.58%
	K28-Side	B	M99-Main	heavy	CDR3	36.80%
	E22-Side	A	G57-Main	light	framework	36.51%
	I31-Main	B	S56-Main	light	framework	40.80%
	E22-Side	A	K60-Side	light	framework	44.04%
	G33-Main	B	Q54-Main	light	framework	58.89%
	K28-Side	B	E102-Side	heavy	CDR3	79.42%
Tetramer by Streltsov et al.	N27-Main	B	R96-Side	light	CDR3	2.72%
	S26-Side	D	Y49-Side	light	framework	3.83%
	K28-Main	B	R96-Side	light	CDR3	3.87%
	D23-Side	D	T53-Side	light	framework	4.34%
	S26-Side	B	Y32-Side	heavy	CDR1	7.72%
	E22-Side	D	Y49-Side	light	framework	8.99%
	S26-Side	D	R46-Side	light	framework	15.28%
	S26-Side	D	D55-Side	light	framework	15.49%
	D23-Side	D	Y32-Side	light	CDR1	18.19%
	N27-Main	B	M99-Side	heavy	CDR3	19.85%
	V18-Main	B	S31-Side	heavy	CDR1	44.43%
	N27-Main	B	Y32-Side	heavy	CDR1	92.94%
	N27-Side	B	D100-Side	heavy	CDR3	94.57%
	N27-Side	B	E102-Side	heavy	CDR3	96.02%
	K28-Side	D	E102-Side	heavy	CDR3	99.50%
octadecamer by Gu et al.	S26-Side	P	Y33-Side	heavy	CDR1	10.53%
	N27-Main	B	S31-Side	heavy	CDR1	11.52%
	S26-Main	F	R96-Side	light	CDR3	13.46%
	N27-Main	P	Q27-Side	light	CDR1	13.53%
	N27-Side	P	Y94-Main	light	CDR3	15.82%
	N27-Side	P	N93-Side	light	CDR3	16.46%
	S26-Side	F	D100-Side	heavy	CDR3	18.99%
	N27-Side	P	N93-Side	light	CDR3	20.42%
	N27-Side	P	P95-Main	light	CDR3	20.60%
	S26-Side	F	R96-Side	light	CDR3	25.86%
	N27-Main	P	Y94-Main	light	CDR3	37.18%

Table S4. The residues forming hydrogen bonds between Fv5E3 and the experimental models of AβOs.
