# Supplementary material for: Molecular interactions between monoclonal oligomer-specific antibody 5E3 and its amyloid beta cognates
Source: PLoS One. 2020 May 29;15(5):e0232266. doi: 10.1371/journal.pone.0232266 (PMC7259632; doi:10.1371/journal.pone.0232266)
Supplement: S5 Table — (PDF) [file pone.0232266.s017.pdf]

	The oligomer residue	The oligomer chain	Fv5E3 residue	Fv5E3 chain	Fv5E3 residue position	Type	Occupancy
Trimer by Kreutzer et al.	I32	B	Y49	light	framework	hydrophobic	46.51%
	D23	B	R46	light	framework	ionic	10.92%
	E22	A	K60	light	framework	ionic	45.56%
	K28	B	E102	heavy	CDR3	ionic	87%
Tetramer by Streltsov et al.	V24	D	Y32	light	CDR1	hydrophobic	33.15%
	V24	D	Y91	light	CDR3	hydrophobic	44.58%
	F19	B	Y94	light	CDR3	hydrophobic	64.14%
	V24	D	A50	light	CDR2	hydrophobic	71.24%
	F19	B	Y32	heavy	CDR1	hydrophobic	74.53%
	K28	D	E102	heavy	CDR3	ionic	99.88%
	F19	B	Y32	heavy	CDR1	aromatic-aromatic	44.42%
	F19	B	Y94	light	CDR3	aromatic-aromatic	56.36%
Octadecamer by Gu et al.	V24	R	A50	light	CDR2	hydrophobic	7.19%
	V24	F	Y94	light	CDR3	hydrophobic	30.47%

Table S5. The residues participating in hydrophobic, ionic, and aromatic-aromatic interactions between Fv5E3 and the experimental models of AβOs.
