# Supplementary material for: Molecular interactions between monoclonal oligomer-specific antibody 5E3 and its amyloid beta cognates
Source: PLoS One. 2020 May 29;15(5):e0232266. doi: 10.1371/journal.pone.0232266 (PMC7259632; doi:10.1371/journal.pone.0232266)
Supplement: S6 Table — (PDF) [file pone.0232266.s018.pdf]

	The oligomer residue	The oligomer chain	Fv5E3 residue	Fv5E3 chain	Fv5E3 residue position	Occupancy
Hexamer by Shafrir et al.	Y27-Side	A	D1-Side	light	framework	7.05%
	A30-Main	B	Y32-Side	light	CDR1	7.35%
	G29-Main	B	Y91-Side	light	CDR3	7.95%
	K28-Side	A	Y27-Main	heavy	CDR1	8.77%
	N27-Main	D	N55-Side	heavy	CDR2	11.60%
	A2-Main	D	N55-Side	heavy	CDR2	14.44%
	N27-Side	F	E28-Main	light	CDR1	15.08%
	K28-Main	A	S31-Side	heavy	CDR1	17.62%
	K28-Main	F	Q27-Side	light	CDR1	18.44%
	D1-Main	D	N77-Side	heavy	framework	18.52%
	N27-Main	B	R96-Side	light	CDR3	20.84%
	K28-Side	D	Y94-Main	light	CDR3	25.78%
	G29-Main	F	Y94-Main	light	CDR3	27.98%
	N27-Main	D	N57-Side	heavy	CDR2	30.43%
	A30-Main	D	S31-Side	heavy	CDR1	30.98%
	N27-Main	D	Y33-Side	heavy	CDR1	41.21%
	K28-Side	B	E102-Side	heavy	CDR3	86.12%
	D1-Side	B	R46-Side	light	framework	95.40%
Hexamer by Laganowsky et al.	I31-Main	D	Y32-Side	light	CDR1	10.61%
	M35-Main	F	Y91-Side	light	CDR3	12.91%
	K28-Side	B	E102-Side	heavy	CDR3	14.46%
	N27-Side	D	E28-Side	light	CDR1	15.50%
	L34-Main	C	G92-Main	light	CDR3	17.87%
	G38-Main	E	S31-Side	heavy	CDR1	20.36%
	K28-Side	D	E28-Side	light	CDR1	20.70%
	V40-Main	A	S30-Main	light	CDR1	22.50%
	K28-Main	D	S30-Side	light	CDR1	22.89%
	M35-Main	F	Y32-Side	heavy	CDR1	23.57%
	V39-Main	A	S30-Side	light	CDR1	27.09%
	V40-Side	F	R98-Side	heavy	CDR3	28.37%
	G33-Main	C	Y32-Side	light	CDR1	42.06%
Dodecamer by Gallion	V24-Main	U	R46-Side	light	framework	13.079%
	K28-Side	U	S56-Side	light	framework	13.58%
	A30-Main	D	G26-Main	heavy	CDR1	13.87%
	I31-Main	D	G26-Main	heavy	CDR1	16.87%
	K28-Main	D	I28-Main	heavy	CDR1	20.52%
	A30-Main	D	I28-Main	heavy	CDR1	21.95%
	N27-Main	D	S31-Side	heavy	CDR1	23.32%
	K28-Side	U	S56-Main	light	framework	25.53%
	F20-Main	D	Y32-Side	heavy	CDR1	27.33%
	S26-Side	U	R46-Side	light	framework	30.01%
	K28-Side	U	D55-Side	light	framework	36.00%
	V18-Main	D	E102-Side	heavy	CDR3	50.61%
	S26-Side	U	D55-Side	light	framework	52.05%
	I32-Main	D	G26-Main	heavy	CDR1	53.35%
	G33-Main	D	G26-Main	heavy	CDR1	54.93%
	D23-Side	U	R46-Side	light	framework	81.46%
	E22-Side	D	R96-Side	light	CDR3	85.02%

Table S6. The residues forming hydrogen bonds between Fv5E3, and the computational and theoretical models of AβOs.
