# Supplementary material for: Molecular interactions between monoclonal oligomer-specific antibody 5E3 and its amyloid beta cognates
Source: PLoS One. 2020 May 29;15(5):e0232266. doi: 10.1371/journal.pone.0232266 (PMC7259632; doi:10.1371/journal.pone.0232266)
Supplement: S7 Table — (PDF) [file pone.0232266.s019.pdf]

	The oligomer residue	The oligomer chain	Fv5E3 residue	Fv5E3 chain	Fv5E3 residue position	Type	Occupancy
Hexamer by Shafrir et al.	I32	B	Y32	light	CDR1	hydrophobic	4.38%
	A2	B	Y49	light	framework	hydrophobic	7.27%
	A30	B	Y49	light	framework	hydrophobic	11.30%
	I31	B	Y32	light	CDR1	hydrophobic	11.36%
	A30	F	I29	light	CDR1	hydrophobic	12.70%
	I31	B	Y91	light	CDR3	hydrophobic	28.01%
	A30	E	Y52	heavy	CDR2	hydrophobic	78.07%
	I31	G	Y94	light	CDR3	hydrophobic	89.13%
	E3	B	K60	light	framework	ionic	3.61%
	K28	B	E102	heavy	CDR3	ionic	95.57%
	K28	A	Y32	heavy	CDR1	cation-π	6.98%
Hexamer by Laganowsky et al.	V40	E	I28	heavy	CDR1	hydrophobic	3.22%
	V40	F	Y52	heavy	CDR2	hydrophobic	3.4%
	V36	G	M99	heavy	CDR3	hydrophobic	5.27%
	V40	F	P53	heavy	CDR2	hydrophobic	6.75%
	L34	D	A51	light	CDR2	hydrophobic	8.01%
	V40	F	Y33	heavy	CDR1	hydrophobic	12.28%
	V36	G	Y32	heavy	CDR1	hydrophobic	12.73%
	V39	F	F29	heavy	CDR1	hydrophobic	13.69%
	A30	B	Y94	light	CDR3	hydrophobic	21.35%
	L34	G	Y32	heavy	CDR1	hydrophobic	23.57%
	L34	G	Y91	light	CDR3	hydrophobic	24.61%
	V40	F	F29	heavy	CDR1	hydrophobic	25.41%
	L34	D	Y32	light	CDR1	hydrophobic	41.93%
Dodecamer by Gallion	F19	U	Y91	light	CDR3	hydrophobic	10.22%
	F19	U	I29	light	CDR1	hydrophobic	11.53%
	I32	D	I28	heavy	CDR1	hydrophobic	14.55%
	F19	U	Y32	light	CDR1	hydrophobic	27.93%
	V18	D	M99	heavy	CDR3	hydrophobic	28.85%
	A21	U	Y91	light	CDR3	hydrophobic	39.82%
	V40	D	Y27	heavy	CDR1	hydrophobic	43.13%
	I31	D	Y27	heavy	CDR1	hydrophobic	54.35%
	K28	U	D55	light	framework	ionic	45.02%
	E22	D	R96	light	CDR3	ionic	85.7%
	D23	U	R46	light	framework	ionic	90.54%
	F19	U	Y91	light	CDR3	aromatic-aromatic	37.91%

Table S7. The residues participating in hydrophobic, ionic and cation-π interactions between Fv5E3, and the computational and theoretical models of AβOs.
