# Supplementary material for: Molecular interactions between monoclonal oligomer-specific antibody 5E3 and its amyloid beta cognates
Source: PLoS One. 2020 May 29;15(5):e0232266. doi: 10.1371/journal.pone.0232266 (PMC7259632; doi:10.1371/journal.pone.0232266)
Supplement: S8 Table — (PDF) [file pone.0232266.s020.pdf]

	The fibril residue	The fibril chain	Fv5E3 residue	Fv5E3 chain	Fv5E3 residue position	Occupancy
The fibril by Lu et al.	D23-Side	V	N55-Side	heavy	CDR2	34.70%
	R5-Side	V	S52-Side	light	CDR2	35.49%
	V40-Side	V	R46-Side	light	framework	35.70%
	D23-Side	V	K59-Side	heavy	framework	36.94%
	V40-Side	V	Y91-Side	light	CDR3	37.89%
	Q15-Side	V	Y91-Main	light	CDR3	42.06%
	R5-Main	V	K60-Side	light	framework	43.13%
	E22-Side	V	K59-Side	heavy	framework	45.96%
	H14-Main	V	S30-Main	light	CDR1	52.36%
	D7-Side	V	K60-Side	light	framework	56.90%
	M35-Main	V	S31-Side	heavy	CDR1	59.59%
	D7-Side	V	S52-Side	light	CDR2	66.13%
	K16-Main	V	G92-Main	light	CDR3	68.07%
	E22-Side	V	R50-Side	heavy	framework	75.17%
	E11-Side	V	S30-Side	light	CDR1	81.48%
	E11-Side	V	R66-Side	light	framework	83.60%
	D23-Side	V	Y33-Side	heavy	CDR1	92.34%
	D23-Side	V	R50-Side	heavy	framework	94.03%
The fibril by Petkova et al.	D23-Main	N	N77-Side	heavy	framework	14.68%
	K16-Side	E	Y91-Side	light	CDR3	14.89%
	N27-Side	N	S31-Side	heavy	CDR1	15.16%
	G19-Main	E	K59-Side	heavy	framework	15.35%
	Q15-Main	E	Y91-Side	light	CDR3	21.97%
	E11-Side	E	K59-Side	heavy	framework	22.36%
	S26-Main	N	T30-Side	heavy	CDR1	23.48%
	V40-Side	E	Y32-Side	heavy	CDR1	24.28%
	Y10-Side	N	V58-Main	light	framework	24.90%
	F19-Main	N	G26-Main	heavy	CDR1	26.74%
	E11-Side	N	Y49-Side	light	CDR3	27.49%
	Y10-Side	E	D61-Main	heavy	framework	31.12%
	G9-Main	N	S52-Main	light	CDR2	31.52%
	E22-Side	E	R66-Side	light	framework	37.35%
	H13-Side	E	S31-Side	heavy	CDR1	38.19%
	G9-Main	N	Q54-Main	light	framework	38.76%
	H14-Side	E	Y91-Side	light	CDR3	47.98%
	E11-Main	E	Y33-Side	heavy	CDR1	48.17%
	K16-Side	E	E28-Side	light	CDR1	61.50%
	L17-Main	E	Y32-Side	light	CDR1	73.46%
	H13-Main	E	Y33-Side	heavy	CDR1	77.48%
	E11-Side	E	Y52-Side	heavy	CDR2	87.66%
	Y10-Side	E	D61-Side	heavy	framework	95.52%
The fibril by Schmidt et al.	S10-Main	A	Q5-Side	heavy	framework	11.03%
	S10-Side	A	N77-Side	heavy	framework	13.14%
	N11-Side	A	G26-Main	heavy	CDR1	14.72%
	N11-Side	A	S7-Side	heavy	framework	14.73%
	N11-Side	M	Y52-Side	heavy	CDR2	18.33%
	V8-Main	A	Q5-Side	heavy	framework	19.67%
	D7-Side	A	V2-Main	heavy	framework	20.56%
	N11-Side	A	A24-Main	heavy	framework	20.67%
	N11-Side	A	Q5-Main	heavy	framework	21.12%
	G9-Main	A	Q6-Main	heavy	framework	22.53%
	N11-Side	A	S7-Main	heavy	framework	26.27%
	S10-Side	A	S76-Main	heavy	framework	29.45%
	G9-Main	A	S76-Main	heavy	framework	29.76%
	N11-Main	A	S7-Side	heavy	framework	34.44%
	N11-Side	M	N55-Side	heavy	CDR2	35.46%
	L18-Main	M	N77-Side	heavy	framework	37.64%
	A14-Main	M	N55-Side	heavy	CDR2	39.46%
	D7-Side	A	Q1-Main	heavy	framework	52.10%
	I16-Main	A	G26-Main	heavy	CDR1	58.27%
	I16-Main	A	I28-Main	heavy	CDR1	76.78%
	V20-Main	A	S31-Side	heavy	CDR1	81.65%
	D7-Side	M	R96-Side	light	CDR3	98.35%

Table S8. The residues forming hydrogen bonds between Fv5E3 and the models of Aβ fibrils.
