# Supplementary material for: Molecular interactions between monoclonal oligomer-specific antibody 5E3 and its amyloid beta cognates
Source: PLoS One. 2020 May 29;15(5):e0232266. doi: 10.1371/journal.pone.0232266 (PMC7259632; doi:10.1371/journal.pone.0232266)
Supplement: S9 Table — (PDF) [file pone.0232266.s021.pdf]

	The fibril residue	The fibril chain	Fv5E3 residue	Fv5E3 chain	Fv5E3 residue position	Type	Occupancy
The fibril by Lu et al.	L34	T	I28	heavy	CDR1	hydrophobic	1.28%
	V39	V	M99	heavy	CDR3	hydrophobic	7.21%
	L34	V	Y52	heavy	CDR2	hydrophobic	8.49%
	V39	V	Y32	heavy	CDR1	hydrophobic	12.57%
	V36	V	Y94	light	CDR3	hydrophobic	20.27%
	M35	V	Y32	heavy	CDR1	hydrophobic	24.42%
	V40	W	I28	heavy	CDR1	hydrophobic	27.99%
	V40	V	Y49	light	framework	hydrophobic	53.9%
	L17	V	Y94	light	CDR3	hydrophobic	70.16%
	F19	V	Y94	light	CDR3	hydrophobic	83.75%
	D23	V	K59	heavy	CDR2	ionic	49.43%
	E22	V	K59	heavy	CDR2	ionic	69.79%
	E11	V	R66	light	framework	ionic	85.59%
	E22	V	R50	heavy	CDR2	ionic	88.93%
	D23	V	R50	heavy	CDR2	ionic	96.31%
	F19	V	Y94	light	CDR3	aromatic-aromatics	24.22%
The fibril by Petkova et al.	F19	E	A51	light	CDR2	hydrophobic	4.37%
	L17	N	I28	heavy	CDR1	hydrophobic	17.82%
	V24	N	I28	heavy	CDR1	hydrophobic	23.22%
	Y10	E	Y52	heavy	CDR2	hydrophobic	25.59%
	F19	N	I28	heavy	CDR1	hydrophobic	30.18%
	L17	E	Y32	light	CDR1	hydrophobic	44.58%
	V12	E	Y94	light	CDR3	hydrophobic	60.08%
	E11	E	R50	heavy	CDR2	ionic	0.17%
	K16	D	E28	light	CDR1	ionic	15.04%
	K16	E	E28	light	CDR1	ionic	63.5%
	E11	E	K59	heavy	CDR2	ionic	87.92%
	Y10	E	Y52	heavy	CDR2	aromatic-aromatic	5.81 %
	K16	E	Y91	light	CDR3	cation-π	89.5%
	Y10	E	R50	heavy	CDR2	cation-π	95.19%
The fibril by Schmidt et al.	V8	A	V2	heavy	framework	hydrophobic	0.09%
	V8	A	L4	heavy	framework	hydrophobic	1.13%
	F3	K	Y32	light	CDR1	hydrophobic	12.42%
	F3	J	Y32	light	CDR1	hydrophobic	17.05%
	L18	A	Y32	heavy	CDR1	hydrophobic	33.33%
	I16	A	Y27	heavy	CDR1	hydrophobic	35.77%
	I15	A	I28	heavy	CDR1	hydrophobic	41.52%
	M19	A	I28	heavy	CDR1	hydrophobic	66.42%
	D7	M	R96	light	CDR3	ionic	99.54%
	F3	K	Y32	light	CDR1	aromatic-aromatic	41.1%

Table S9. The residues participating in hydrophobic, ionic and aromatic-aromatic interactions between Fv5E3 and the models of Aβ fibrils.
