# Supplementary material for: Molecular interactions between monoclonal oligomer-specific antibody 5E3 and its amyloid beta cognates
Source: PLoS One. 2020 May 29;15(5):e0232266. doi: 10.1371/journal.pone.0232266 (PMC7259632; doi:10.1371/journal.pone.0232266)
Supplement: S10 Table — (PDF) [file pone.0232266.s022.pdf]

	The cross-β subunit residue	The cross-β subunit chain	Fv5E3 residue	Fv5E3 chain	Fv5E3 residue position	Occupancy
The model of cross-β sub-unit by Lührs et al.	E22-Side	A	Y52-Side	heavy	CDR2	10.32%
	S26-Side	A	S30-Main	light	CDR1	10.67%
	S26-Side	F	G92-Main	light	CDR3	12.01%
	E22-Side	F	R50-Side	heavy	framework	12.68%
	E22-Side	B	K59-Side	heavy	framework	14.43%
	S26-Side	F	N93-Side	light	CDR3	15.65%
	N27-Side	A	S30-Side	light	CDR1	16.71%
	N27-Side	B	S30-Side	light	CDR1	21.45%
	S26-Main	B	Y94-Side	light	CDR3	23.47%
	V24-Main	A	Y91-Side	light	CDR3	24.99%
	S26-Main	B	G92-Main	light	CDR3	25.56%
	S26-Main	F	Y94-Side	light	CDR3	32.90%
	S26-Side	F	Y94-Side	light	CDR3	35.71%
	E22-Side	B	R50-Side	heavy	framework	61.14%
	E22-Side	D	K59-Side	heavy	framework	64.21%
	E22-Side	F	K59-Side	heavy	framework	91.23%
The model of cross-β sub-unit by Xiao et al.	K28-Side	G	Y32-Side	light	CDR1	1.49%
	S26-Side	B	F29-Main	heavy	CDR1	5.14%
	K28-Side	B	E102-Side	heavy	CDR3	6.74%
	K28-Side	F	Y32-Side	light	CDR1	8.03%
	A42-Side	A	Q1-Side	heavy	framework	8.06%
	A42-Side	A	Q1-Main	heavy	framework	8.18%
	S26-Side	B	G26-Main	heavy	CDR1	9.36%
	A42-Side	E	Y32-Side	light	CDR1	15.40%
	S26-Side	B	T30-Main	heavy	CDR1	16.48%
	A42-Side	A	R98-Side	heavy	CDR3	21.99%
	K28-Side	A	G26-Main	heavy	CDR1	22.50%
	S26-Side	B	Y27-Main	heavy	CDR1	35.65%
	K28-Side	B	Y27-Side	heavy	CDR1	48.58%

Table S10. The residues forming hydrogen bonds between Fv5E3 and the cross-β sub-units of Aβ fibrils.
