# Supplementary material for: Molecular interactions between monoclonal oligomer-specific antibody 5E3 and its amyloid beta cognates
Source: PLoS One. 2020 May 29;15(5):e0232266. doi: 10.1371/journal.pone.0232266 (PMC7259632; doi:10.1371/journal.pone.0232266)
Supplement: S11 Table — (PDF) [file pone.0232266.s023.pdf]

	The cross-β subunit residue	The cross-β subunit chain	Fv5E3 residue	Fv5E3 chain	Fv5E3 residue position	type	Occupancy
The cross-β sub-unit by Lührs et al.	V24	E	Y94	light	CDR3	hydrophobic	4.07%
	V18	D	V56	heavy	CDR2	hydrophobic	15.64%
	V24	D	Y94	light	CDR3	hydrophobic	25.95%
	F20	F	V56	heavy	CDR2	hydrophobic	29.52%
	V24	B	G92	light	CDR3	hydrophobic	51.73%
	V24	A	Y91	light	CDR3	hydrophobic	63.59%
	V24	F	Y94	light	CDR3	hydrophobic	79.21%
	E22	F	R50	heavy	framework	ionic	65.51%
	E22	D	K59	heavy	framework	ionic	78.18%
	E22	B	R50	heavy	framework	ionic	87.71%
	E22	F	K59	heavy	framework	ionic	97.46%
The cross-β sub-unit by Xiao et al.	V39	A	V2	heavy	framework	hydrophobic	0.22%
	I31	A	V2	heavy	framework	hydrophobic	2.76%
	I41	A	V2	heavy	framework	hydrophobic	6.18%
	K28	B	E102	heavy	CDR3	ionic	77%
	K28	B	Y27	heavy	CDR1	cation-π	49.92%

Table S11. The residues participating in hydrophobic, ionic, and aromatic-aromatic interactions between Fv5E3 and the cross-β sub-units of Aβ fibrils.
